# IRE1α arm of unfolded protein response in muscle-specific TGF-β signaling-mediated regulation of muscle cell immunological properties

**DOI:** 10.1186/s11658-023-00429-w

**Published:** 2023-02-27

**Authors:** Jiangwei Xiao, Jingwen Huang, Xiaoting Jian, Han Wang, Haiqiang Lan, Zhaohong Liao, Ruicai Gu, Jijie Hu, Hua Liao

**Affiliations:** 1 Guangdong Provincial Key Laboratory of Construction and Detection in Tissue Engineering, Department of Anatomy, School of Basic Medical Science, Southern Medical University, Guangzhou, 510515 China; 2 Department of Cell Biology, University of Oklahoma Health Science Center, Oklahoma City, OK USA; 3 Department of Orthopaedics and Traumatology, Nanfang Hospital, Southern Medical University, Guangzhou, 510515 China

**Keywords:** Myofiber, Inflammation, TGF-β, UPR, IRE1α, p38 MAPK

## Abstract

Endoplasmic reticulum stress (ERS) and the unfolded protein response (UPR) are involved in various muscle pathological states. The IRE1α arm of UPR can affect immunological properties of myofiber through restraining p38 mitogen-activated protein kinases (MAPK) activation under inflammatory milieu. However, the relevant pathway molecules regulating the initiation of the IRE1α arm in myofiber remain unclear. In this work, expression of transforming growth factor-beta (TGF-β) and TGF-β receptor II (TGF-βr2), and UPR pathway activation were examined in cardiotoxin (CTX)-damaged mouse muscle, which revealed the activation of TGF-β signaling and UPR in CTX-damaged muscle and in regenerating myofibers. Using control or transgenic mice with TGF-βr2 deleted in skeletal muscle (SM TGF-βr2^−/−^) and the derived primary differentiating myogenic precursor cells (MPCs) treated with/without ERS activator or inhibitor, IRE1α pathway inhibitor, or TGF-β signaling activator, this study further revealed an essential role of intrinsic TGF-β signaling in regulating muscle cell to express inflammation-related molecules including H-2K^b^, H2-Eα, TLR3, and special myokines. TGF-β signaling prompted UPR IRE1α arm and restrained p38 MAPK activation in myofiber under inflammatory milieu. This study uncovers a previously unrecognized function of TGF-β signaling acting as an upstream factor controlling myofiber immune capacities in the inflamed state through the UPR–IRE1α–p38 MAPK pathway.

**Supplementary Information:**

The online version contains supplementary material available at 10.1186/s11658-023-00429-w.

## Background

In various mammalian cell types, endoplasmic reticulum (ER) function could be disrupted by diverse environmental and genetic factors, causing misfolded and unfold proteins to accumulate in ER lumen, eventually leading to ER stress (ERS). To respond to this stress and restore cell homeostasis, the unfolded protein response (UPR), a signal transduction network, could be activated by generated ERS [1–3]. Interestingly, the network of ER, called sarcoplasmic reticulum in skeletal muscle, is extremely extensive and plays a pivotal role in adjusting calcium homeostasis and proteostasis of skeletal muscle. Meanwhile, there is evidence that the UPR response broadly maintains the homeostasis of muscle stem cells, regulates myogenic differentiation, and repairs the damaged muscle [4–7]. In addition, in diverse physiological and pathological processes of skeletal muscle, ERS and UPR markers can be readily detected. For instance, the UPR response is activated after consumption of a high-fat diet, resulting in increased expression of inositol-requiring enzyme-1α (IRE1α) and immunoglobulin heavy chain-binding protein (BiP) in tibialis anterior and soleus muscles [8]. Activation of ERS was also found in skeletal muscle following acute stress induced by long distance running, accompanied by enhanced expression of spliced X box-binding protein-1s (XBP-1s) and Bip [9]. In addition, the protein kinase R (PKR)-like endoplasmic reticulum kinase (PERK) arm of UPR has been reported to prevent loss of muscle mass and strength caused by cancer cachexia [10, 11]. Thus, the UPR is one of the responses that helps skeletal muscle maintain homeostasis in a changing environment.

The ERS plays a pivotal role in skeletal muscle diseases involving polymyositis (PM), dermatomyositis (DM), and sporadic inclusion-body myositis (s-IBM). Meanwhile, reports have shown that the ERS was involved in regulating muscle inflammation. In response to inflammatory stimuli, myocytes could express immunological molecules to obtain immunological properties, which help them participate in the muscle immune response or inflammation [12–14]. Previously, a related study explored the effects of ERS on the intrinsic immunological capacities of myofibers in inflammatory milieu, and showed that the UPR arm of IRE1α plays an important role in adjusting the expression of immunobiological molecules in myofibers through inhibiting p38 MAPK activity [15]. However, further research is needed to explore the regulatory mechanism of UPR pathway activation in myofibers under a proinflammatory environment.

During muscle inflammation and the repair/remodeling process, transforming growth factor-beta (TGF-β) promotes the proliferation, differentiation, and fusion of muscle satellite cells into multinucleated muscle fibers. In addition, it is an important initiating factor of extracellular matrix (ECM) progressive deposition and tissue fibrosis [16–22]. TGF-β secreted by macrophages reflects the transformation of macrophages from an inflammatory phenotype to an antiinflammatory phenotype at injury muscle after phagocytosis of necrotic or apoptotic cells, which appears to be part of the mechanism for addressing muscle inflammation [20, 21, 23]. It has been reported that TGF-β signaling can control muscle immune function by directly regulating inflammatory molecules, such as intercellular cell adhesion molecule-1 (ICAM-1) [23], human leukocyte antigen class I (HLA I) [24], and interleukin-6 (IL-6) secretion in myofibers [25]. We recently found TGF signaling elevated in regenerated myofibers and affected the muscle inflammation response [26]. A question may arise as to whether myofiber-specific TGF-β signaling plays a role in the development of local inflammation after myoinjury, through regulating myofiber ERS and UPR response.

The present study addressed this question and observed that TGF-β signaling and UPR were activated in CTX-damaged muscles and in regenerating myofibers in mice. Furthermore, using a myoinjury model in mice with muscle-specific TGF-β receptor 2-deficiency (SM TGF-βr2^−/−^), this study has revealed an essential role of intrinsic TGF-β signaling in muscle cell immunological capacities through prompting the activity of the IRE1α arm of UPR under inflammatory stimulus.

## Methods

### Mice

C57BL/6 (B6) mice were provided by the Animal Experimentation Centre of the Southern Medical University. Mice with TGF-βr2 knockout in skeletal muscle (designated as SM TGF-βr2^−/−^ mice, KO mice) were obtained by crossing TGF-βr2^flox/flox^ mice with MCK-Cre mice, which were purchased from the Jackson Laboratory. A polymerase chain reaction (PCR) of mouse tail DNA was used to determine the genotypes of generated mice [26]. TGF-βr2^flox/flox^ mice were used as the control for KO mice.

### Animal experiments

The total of 50 μl cardiotoxin (CTX) solution (50 μg/ml, Sigma, USA) was used to prepare mice acute injury to unilateral tibialis anterior (TA) muscle [27, 28]. For examining the changes of the related genes and proteins, the damaged TA muscle samples were collected after 0, 4, 7, 10, 15, or 30 days of injection. For histological analysis, muscle samples were frozen in cooled isopentane.

### Cell cultures

The limb muscles of neonatal TGF-βr2^flox/flox^ or SM TGF-βr2^−/−^ mice were used to collect murine myogenic precursor cells (MPCs) in a sterile condition. The dissociated muscles were washed twice with phosphate-buffered saline (PBS) and cut with scissors to obtain smaller chunks, then digested by Collagenase II (Sigma, USA). After digestion, the muscle homogeneous slurry was filtered and centrifuged for preparing the single-cell suspension. Mice Satellite Cell Isolation Kit (Miltenyi Biotec, Germany) was used to isolate MPCs. In brief, the isolated cells were resuspended, treated with Enzyme A, incubated, and then Satellite Cell Isolation solution was added. Cell suspension was applied onto the LS column, placed in the magnetic field of a suitable MACS Separator (Miltenyi Biotec, Germany), and flow-through containing unlabeled cells, representing the enriched satellite cells, was collected. The collected cells were then cultured in growth medium (adding 10% fetal bovine serum). When the cultured MPCs covered 70–80% of the cell culture dish area, the growth medium was substituted by a differential medium (adding 2% horse serum) for 72 h to differentiate the cells into myotubes (MPC-myotubes). For proinflammatory stimuli, lipopolysaccharide (LPS, 100 ng/ml, R&D Systems, USA) and IFN-γ (3 ng/ml, R&D Systems, USA) were added to the fresh medium. To inhibit UPR, MPC-myotubes were treated with 4-phenyl butyric acid (4-PBA) (10 mM, UPR inhibitor, Selleck, Shanghai, China), 4μ8c (50 mM, IRE1α pathway inhibitor, Selleck), or GSK2606414 (1 mM, PERK pathway inhibitor, which can directly bind to PERK and inhibits its activity, Selleck) for about 4 h [15, 29]. For activating UPR, MPC-myotubes were treated with tunicamycin (TM, 1 mg/ml, Santa Cruz California, USA) or thapsigargin (Tg, 0.2 mmol/l, Santa Cruz) for about 4 h [15, 30, 31]. For blocking p38 activity, MPC-myotubes were cultured with SB202190 (20 mM, Sigma) for 4 h [15, 32]. For re-activating the TGF-β/Smad signaling pathway, MPC-myotubes were cultured with SRI-011381 hydrochloride (SRI, 8 μg/ml, MedChemExpress, USA) for about 4 h [33].

### Transcriptome microarray

TRIzol (Life Technologies, USA) was used to extract total RNA from TA muscle. RNA was used to analyze the global transcriptional expression using a commercial kit (G4140-90040, Aglilent Technologies). In brief, the extracted RNA was amplified and labeled with CY-3 using a kit (G4140-90040, Aglilent Technologies). After labeling, the RNA was hybridized according to the GE Hybridization Kit protocol. Then, the Agilent Technologies Scanner and Feature Extraction was used to scan arrays and extract spot intensities and other quality control features. Agilent control features and a spike-in control were used to assess the array quality and then the signals were processed. All arrays were automatically normalized by Agilent’s Feature Extraction Software; the background and outlier spots that included saturation or nonuniformity were subtracted and flagged. More details about the software can be found at http://www.chem.agilent.com. Finally, the Feature Extraction Software (Aglilent Technologied) was used to analyze the results, with all values being averaged from four independent experiments.

### RNA extraction and real-time quantitative PCR assay

Total RNA was extracted from muscle samples or primary MPCs by TRIzol, and then converted into cDNA using a commercial kit (k1622, Thermo Fisher Scientific, USA). Real-time quantitative PCR assays were used to detect the mRNA levels of TGF-β2, TGF-βr2, IL-1β, IL-6, MCP-1, C/EBP-homologous protein (CHOP), XBP-1s, activating transcription factor 6 (ATF6), ATF4, and eukaryotic initiation factor 2α (eIF2α). All genes were analyzed and computed against GAPDH. Primer sequences for PCR are described in Table1. The 2^−ΔΔCt^ method was used for determining fold change and calculating the relative expression of the genes. The Ct of target genes were normalized to the Ct of GAPDH.

**Table 1:** Primer sequences used for PCR

Genes	Sequences (5′–3′)
TGF-β2	Forward: GGCGGTGCTCGCTTTGAT Reverse: TCCCGAATGTCTGACGTATTGA
TGF-βr2	Forward: GTGAGACTGTCCACTTGCGA Reverse: TGTCGTTCTTCCTCCACACG
CHOP	Forward: GCATGAAGGAGAAGGAGCAG Reverse: CTTCCGGAGAGACAGACAGG
Xbp-1s	Forward: GAGTCCGCAGCAGGTG Reverse: GTGTCAGAGTCCATGGGA
ATF6	Forward: TGATGGCTGTCCAGTACACA Reverse: GCAGATGATCCCTTCGAAAT
ATF4	Forward: CCTTGTAAGACACCGGAAAT Reverse: TAGAGATCGTCCTAAAGGC
eIF2α	Forward: AATCAATGTCGCTAACAAGG Reverse: TAAAGTTGTAGGTTAGGCGT
IL-1β	Forward: CAGCGACAGAGCCAGAAT Reverse: AGGGACGGAAAGTGGAAC
IL-6	Forward: GAGTCCGCAGCAGGTG Reverse: GTGTCAGAGTCCATGGGA
MIP-1α	Forward: CCATGGGTCCCGTGTAGAGC Reverse: TGAAGAGTCCCTCGATGTGGC
MCP-1	Forward: TGATGGCTGTCCAGTACACA Reverse: GCAGATGATCCCTTCGAAAT
GAPDH	Forward: CAATGTGTCCGTCGTGGATCT Reverse: GTCCTCAGTGTAGCCCAAGATG

### Histological and immunofluorescence detection

Cultured MPC-myotubes or muscle cryosections (8 μm thickness) were collected for hematoxylin and eosin (H&E) or immunostaining. To perform immunofluorescence assays, cold acetone was first used to fix the samples, followed by incubation with primary antibodies overnight at 4 °C. Antibodies used included rat anti-mouse F4/80 (1:250, eBioscience, 56-4801-80), rabbit anti-mouse IRE1α (p Ser724) (1:250, NOVUS, NB100-2323), mouse anti-mouse eIF2α (p S51) (1:250, Abcam, ab32157), mouse anti-mouse ATF6 (1:250, NOVUS, NBP1-40256), rat anti-mouse CD11b (1:250, eBioscience, 50-0112-82), rabbit anti-mouse dystrophin (1:500, Bioss, bs-14477R), rat anti-mouse laminin (1:200, Abcam, ab11576), rabbit polyclonal anti-fast myosin muscle heavy chain (MyHC, 1:500, abcam, ab91506), rabbit anti-mouse myogenin (1:500, Invitrogen, PA5-116750), rabbit anti-mouse desmin (1:500, abcam, ab32362), rabbit polyclonal anti-myosin-3 (1:500, bs-10905R, bioss), and mouse anti-mouse p38 (1:500, Santa Cruz, sc-166182). The next day, the samples were incubated with goat anti-rat IgG H&L (FITC) (1:500, Abcam, ab6840), Cy3 conjugated goat anti-rat IgG, Cy3 conjugated goat anti-rabbit IgG, Alexa Fluor 488 conjugates goat anti-mouse IgG, or Alexa Fluor 488 conjugates goat anti-rabbit IgG (1:500, Beyotime, A0507, A0516, A0428, A0423). Finally, DAPI was used for counterstaining cell nuclei. An Olympus BX51 fluorescence microscope (Olympus, Japan) was used to analyze the muscle sections or cultured MPCs. For measuring the area ratio of the target protein, more than six images were randomly selected, then the total area of positive protein in the full field of each image and the total area of each image were measured using Image-Pro Plus software (IPP, Media Cybernetics, USA). The area ratio of positive protein [(the total positive area/the total area of each image) × 100%] was calculated. For measuring the intensity of fluorescent staining, the full-image integrated optical density (IOD) and the area of interest (AOI) of all the positive stains were measured in more than six randomly selected images using IPP software. The mean fluorescence staining intensity [(IOD/AOI) × 100%] was then calculated. For measurement of the myofiber cross-sectional area (CSA), 11 randomly selected images were used. First, the total 25–50 fibers area per image (total area, pixels^2^) were manually evaluated and calculated by image J software (NIH, USA), then the mean myofiber CSA was calculated as the total area/fiber number.

### Western blot analysis

Muscle tissue and cell proteins were extracted using a protein extraction kit (KeyGEN, Jiangsu, China). The levels of protein expression were examined for TGF-β2, TGF-βr2, phosphorylated IRE1α (P-IRE1α), total IRE1α (T-IRE1α), P- eIF2α, T-eIF2α, ATF6, P-p38, T-p38, phosphorylated extracellular regulated protein kinases 1/2 (P-Erk1/2), T-Erk1/2, phosphorylated c-June N-terminal kinase (P-JNK), T-JNK, P-smad2/3, smad2/3, major histocompatibility complex class I molecule(H-2K^b^), major histocompatibility complex class II molecule (H2-Ea), toll-like receptors-3 (TLR3), P-p65, and T-p65. The following primary antibodies were used: rabbit anti-mouse TGF-β2 (1: 500, Bioss, bs-20412R), rabbit anti-mouse TGF-βr2 (1: 500, Bioss, bs-0117R), mouse anti-mouse TLR3 (1:1000, NOVUS, NBP2-24875), rabbit anti-mouse H-2K^b^ (1:500, Abcam, ab93364), mouse anti-mouse H2-Eα (1:1000, NOVUS, NBP1-43312), rabbit anti-mouse IRE1α (1:1000, NOVUS, NB100-2324), rabbit anti-mouse IRE1α (p Ser724) (1:1500, NOVUS, NB100-2323), mouse anti-mouse eIF2α (1:1000, Abcam, ab5369), rabbit anti-mouse eIF2α (p S51) (1:1000, Abcam, ab32157), mouse anti-mouse ATF6 (1:5000, NOVUS, NBP1-40256), rabbit anti-mouse p65 (1:1000, CST, 8242), rabbit anti-mouse P-p65 (1:1000, CST, 3033), rabbit anti-mouse Erk1/2 (1:1000, CST, 4695), rabbit anti-mouse P-Erk1/2 (1:1000, CST, 4370), mouse anti-mouse p38 (1:1000, Santa Cruz, sc-7972), mouse anti-mouse p38 (1:1000, Santa Cruz, sc-166182), rabbit anti-mouse JNK (1:1000, CST, 9252), rabbit anti-mouse P-JNK (1:1000, CST, 9255), rabbit anti-mouse Smad2/3 (1:500, Abcam, ab217553), and mouse anti-mouse GAPDH (1:5000, Fudebio, FD0063-100). After incubating overnight at 4 °C, the secondary antibodies were added, including goat anti-rabbit IgG-HPR (1:5000, Fudebio, FD0128) or goat anti-mouse IgG-HPR (1:5000, Fudebio, FD0142). The quantification of protein bands was performed using ImageJ v1.42 software (National Institutes of Health, USA). The ratio, protein of interest/GAPDH or phosphorylation/total, was used to express the relative protein level values. Since the p-eIF2α colocalized with DAPI due to the activated PERK phosphorylated eIF2α [34], p-eIF2α protein expression was used to verify PERK arm activation in muscle.

### Luminex assay

After 72 h differentiation, MPC-myotubes were treated with LPS (100 ng/ml) and IFN-γ (3 ng/ml) for approximately 24 h and 48 h. Cultured media were collected and centrifuged to obtain supernatants. The levels of myokines were measured by Luminex xMap technology with Bio-Rad Bio-Lpex 200 apparatus (Bio-Rad Laboratories, Inc., China), which includes Eotaxin, G-CSF, GM-CSF, IFN-γ, IL-1α, IL-1β, IL-10, IL-12p40, IL-12p70, IL-13, IL-17A, IL-3, IL-6, MCP-1, MIP-1α, RANTES, and TNF-α.

### Cell sorting and flow cytometry analysis

Damaged TA muscles were collected and minced, and then gently digested twice with 0.2% II type collagenase (Sigma) at 37 °C for 45 min. Total cells were isolated from muscle homogenate and blocked. The cells were labeled with anti-CD45-Pacific Blue, anti-CD11b-PE, anti-F4/80-PE, anti-Ly6C-FITC, and anti-CD206-eFluor 700. A FACSAria II cell sorter with FlowJo software (BD Biosciences, Franklin Lakes, New Jersey, USA) was used to analyze the labeled cells. All antibodies were validated for specificity, following the instructions.

### Statistical analyses

Quantitative values were shown as mean ± standard deviation. SPSS v. 20.0 (IBM, Armonk, New York, USA) software was used to perform a one-way ANOVA or two-sample *t*-test for multiple or independent comparisons. *P* < 0.05 was set as statistically significant. Additional statistical information is presented in Additional file 1.

## Results

### Upregulation of TGF-β signaling in CTX-injured muscle affects muscle inflammation and muscle cells, producing immune-relevant molecules

**Fig. 1:**
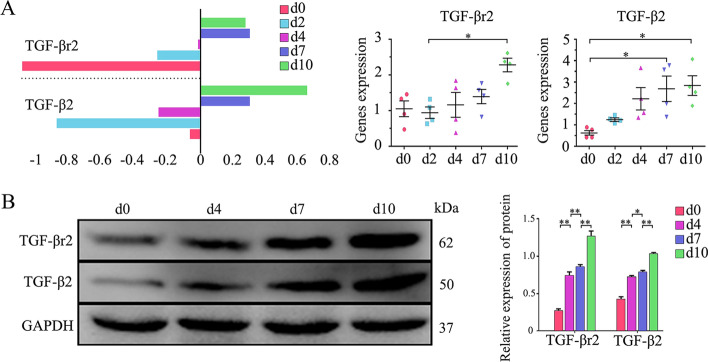
The expression of TGF-β2 and TGF-βr2 in CTX-injured TA muscle. **A** RNA levels of TGF-βr2 and TGF-β2 in WT mice injured TA muscle, analyzed by microarray experiment and qPCR. **B** The protein expression of TGF-βr2 and TGF-β2 in WT mice injured TA muscle, analyzed by Western blot. The relative protein levels are expressed as a ratio (protein of interest/GAPDH). All data are presented as means ± SD (*n* = 3). A one-way ANOVA was used for multiple comparisons (*P < 0.05; **P < 0.01)

As shown in Fig. 1, transcriptome microarray and PCR analyses revealed that wild-type (WT) mice gene expression of TGF-β2 (not of TGF-β1 or TGF-β3) and TGF-βr2 were comparatively lower in normal and 2-d injured TA muscles, but elevated in 4, 7, and 10-d damaged muscles (Fig. 1A). Western blot analyses consistently revealed that from day 4 to day 10 after myoinjury, muscles had a rapid increase in protein expression of TGF-β2 and TGF-βr2 (Fig. 1B), implying the importance of TGF-β signaling in muscle inflammation and regeneration.

**Fig. 2:**
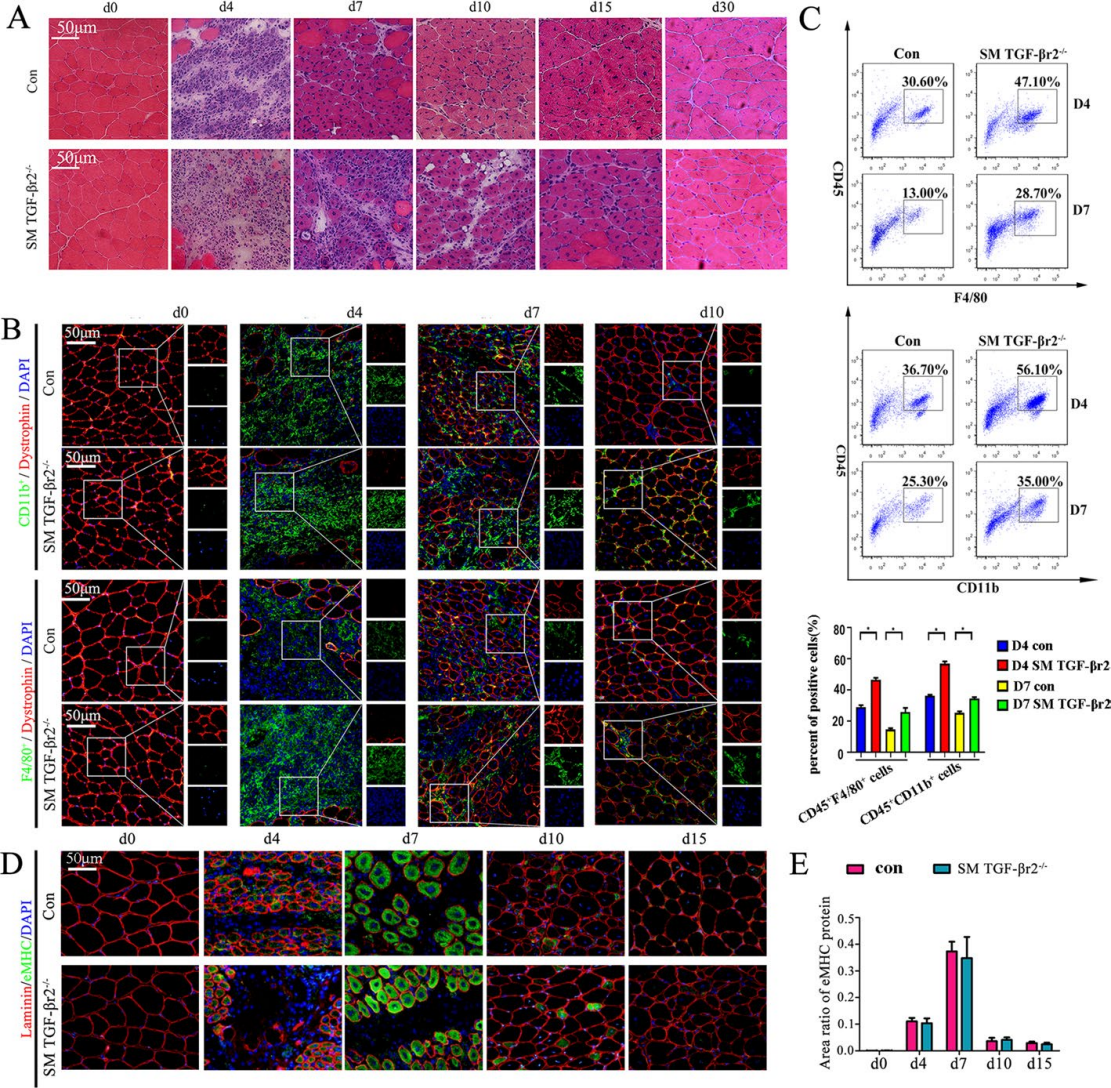
The effect of intrinsic TGF-β signaling on muscle inflammation and regeneration. **A** Histological features of the CTX-damaged TA muscle in control or SM TGF-βr2^−/−^ mice. **B** Representative immunofluorescence staining results of CD11b, F4/80, and dystrophin in damaged TA muscle. **C** FACS analysis of the proportion of CD45^+^CD11b^+^ and CD45^+^F4/80^+  ^cells sorted from TA muscle on days 4 and 7 post-injury. **D**, **E** Embryonic myosin heavy chain (eMHC) staining and the quantitative results in damaged TA muscle. All data are presented as means ± SD (*n* = 3). One-way ANOVA was used for multiple comparisons (*P < 0.05). Bar = 50 μm

Our recent work proved that deficiency of TGF-β signaling in muscle cells caused more serious infiltration of macrophages and CD4 T cells at the degeneration stage (D4) and the early stage of regeneration (D7) after myoinjury [^+^^26^]. To further clarify this, the inflammatory response was evaluated using muscle TGF-βr2 deficient mice (SM TGF-βr2^−/−^ mice), which were hybridized from TGF-βr2-floxed mice and MCK-Cre mice (Additional file 2: Fig. S1) [26]. As shown in Fig. 2A, the inflamed muscle from SM TGF-βr2^−/−^ mice showed a significant increase in inflammatory infiltration than TGF-βr2^flox/flox^ mice on days 4, 7, and 10 post-injury. In agreement, immunostaining and fluorescence-activated cell sorting (FACS) analysis showed that the loss of TGF-β signaling specifically in myofibers resulted in a significant increase in the infiltration of CD11b and F4/80 cells in inflamed muscle (Fig. ^+^^+^2B, C). Further, FACS analysis and PCR demonstrated that in myofibers, TGF-β signaling loss impaired the muscle macrophages transition from M1 (F4/80^+^Ly6C^+^) to M2 phenotype (F4/80^+^CD206^+^) (Additional file ^+^^+^^+^^+^2: Fig. S2A), and elevated gene levels of pro-inflammatory IL-1β, IL-6, MCP-1, and MIP-1α in inflamed muscle (Additional file 2: Fig. S2B). Our results thus uncovered that targeted blocking of myofiber TGF-β signaling aggravated the muscle inflammation response. To determine whether the lack of TGF-β signaling effects the muscle regenerative potential, we performed immune staining for embryonic myosin heavy chain (eMHC), myogenin, and fast muscle myosin heavy chain (MyHC) in inflamed muscle on days 4, 7, 10, and 15 post-myoinjury. No significant expression differences for these myogenic proteins in centronucleated fibers were observed between SM TGF-βr2^−/−^ and control mice (Fig. 2D, E, and Additional file 2: Fig. S3A). Consistently, we did not find marked difference for myofiber cross-sectional area (CSA) between SM TGF-βr2^−/−^ and control mice (Additional file 2: Fig. S3B). It is suggested that myofiber-specific inhibition of TGF-β protects skeletal muscle from acute injury [33]. However, our data suggested myofiber-specific inhibition of TGF-β signaling had no obvious impact on myocyte regeneration.

**Fig. 3:**
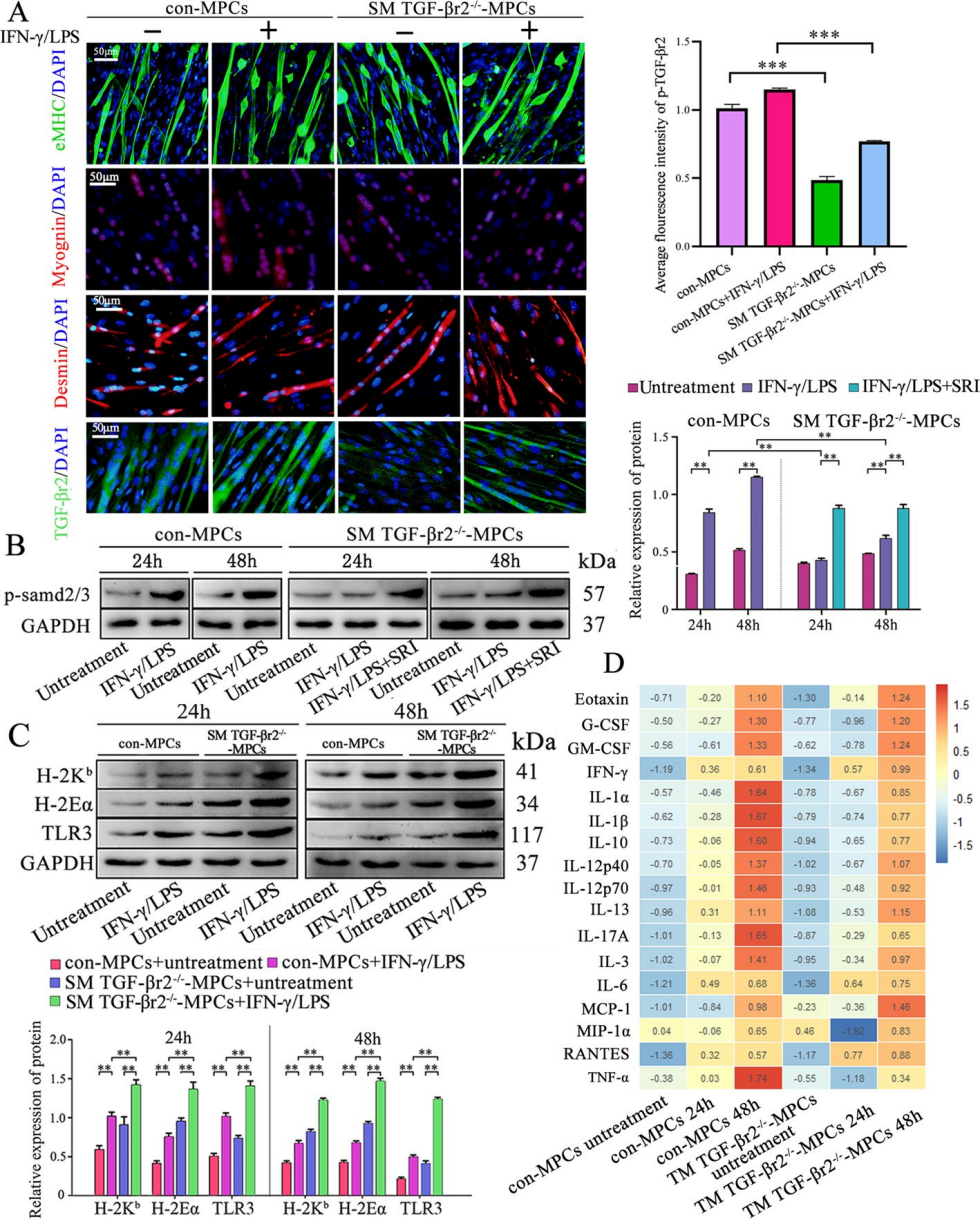
Intrinsic TGF-β signal controls immunological molecules and myokine expression in cultured primary myotubes. **A** Representative immunofluorescence staining for desmin, myogenin, eMHC, and TGF-βr2 in SM TGF-βr2^−/−^ mice-derived MPC-myotubes (MPCs) that received pro-inflammatory stimuli or not. **B**, **C** Western blot analysis of the protein expression of p-Smad2/3, H-2K^b^, H2-Eα, and TLR3 in MPC-myotubes that received pro-inflammatory stimuli or not. **D** Luminex assay of protein level changes for pro-inflammatory myokines in control or SM TGF-βr2^−/−^ mice-derived MPC-myotubes exposed to inflammatory milieu or not. Before creating the heat map, log transformation was used to process the data and plot in R language. The relative protein levels are expressed as a ratio (protein of interest/GAPDH). All data are presented as means ± SD (*n* = 3 replicates). One-way ANOVA was used for multiple comparisons (**P < 0.01; ***P < 0.001). Bar = 50 μm

To further elucidate the mechanisms of how intrinsic TGF-β signaling affects muscle inflammation, SM TGF-βr2^−/−^ or control mice-derived MPCs underwent 72 h HS-differentiation into myotubes (MPC-myotubes). After 24 h pro-inflammatory stimuli, we performed TGF-βr2, myogenin, desmin, and eMHC staining for MPC-myotubes. We observed a decrease in the TGF-βr2 level in KO mice-derived MPC-myotubes compared with control (Fig. 3A). However, deficiency of TGF-β signaling had no effects on muscle cell differentiation (myogenin, eMHC, and desmin) under pro-inflammatory milieu (Fig. 3A). Protein blot analysis further showed a decrease of p-Smad2/3, a key TGF-β pathway molecule in TGF-βr2^−/−^ MPC-myotubes, compared with control MPC-myotubes with pro-inflammatory stimulation (Fig. 3B). When treated with Smad agonist SRI-011381 hydrochloride (SRI), the low expression of p-Smad2/3 in TGF-βr2^−/−^ MPC-myotubes were strongly corrected (Fig. 3B). Since H-2K^b^ and H2-Eα are important molecules for myofiber presenting antigens to T cells [12, 13] and TLR3 has a vital role in skeletal muscle innate immunity [12, 20]. We subsequently conducted Western blot analyses, which showed that pro-inflammatory stimuli induced elevation of TLR3, H2-Eα, and H-2K^b^ proteins in cultured control MPC-myotubes (Fig. 3C). Noteworthy, with stimulation of LPS and IFN-, the expression levels of TLR3, H2-Eα, and H-2K^b^ proteins in TGF-βr2^−/−^ MPC-myotubes were distinctly higher than those in control MPC-myotubes (Fig. 3C), suggesting a role of intrinsic TGF-β signal in the control of expression of myofiber immunological molecules.

Furthermore, the protein levels of muscle-derived cytokines (myokines) in TGF-βr2-present or TGF-βr2-deficient MPC-myotubes were examined by Luminex assays. As expected, pro-inflammatory treatment resulted in an obvious protein level elevation of some myokines in control MPC-myotubes, including eotaxin, G-CSF, GM-CSF, IFN-γ, IL-1α, IL-1β, IL-10, IL-12p40, IL-12p70, IL-13, IL-17A, IL-6, MCP-1, MIP-1α, RANTES, and TNF-α. Of note, in response to IFN-γ/LPS pro-inflammatory stimuli, the lack of TGF-β signal in MPC-myotubes resulted in a further increase of the levels of pro-inflammatory Eotaxin, IFN-γ, IL-6, MCP-1, and RANTES when compared with control MPC-myotubes (Fig. 3D). This was consistent with our finding that the infiltration increase of CD11b and F4/80 cells in inflamed muscle of SM TGF-βr2^+^^+^^−/−^ mice (Fig. 2B, C), since MCP-1 and RANTES (CCL5) are powerful attractors for monocytes and macrophages in peripheral tissue [35]. Together, our work suggests the importance of the muscle-specific TGF-β signaling in local inflammatory cell recruitment.

### Intrinsic TGF-β signaling affects muscle cell immunological characteristics through prompting UPR activity under an inflammatory stimulus

**Fig. 4:**
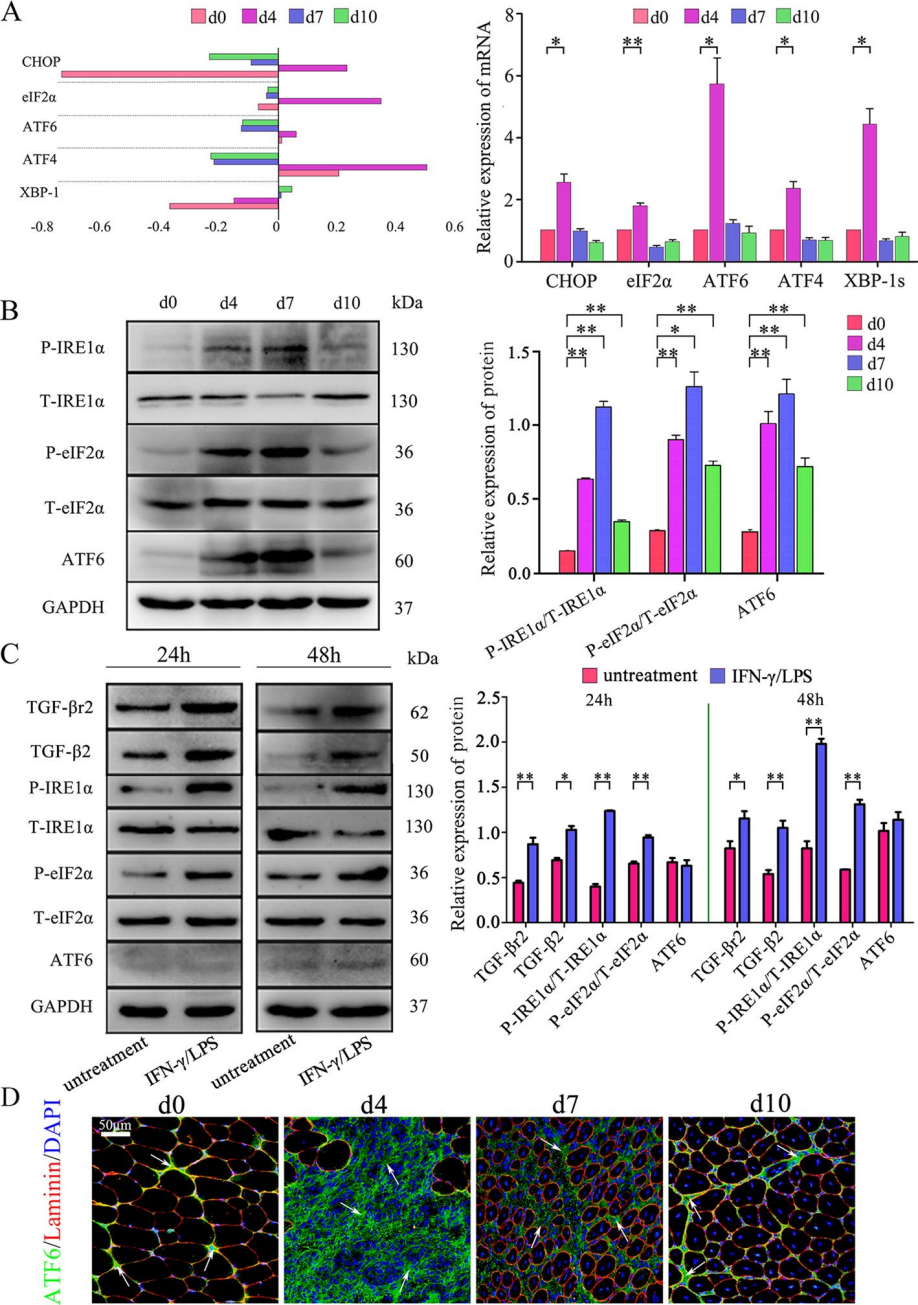
Intrinsic TGF-β signal modulates myofiber UPR activity under the inflammatory stimulus. **A** Gene levels of UPR pathway molecules in WT mice injured TA muscle were analyzed by microarray and q-PCR. **B**, **C** The protein expression of UPR pathway molecules in WT mice injured TA muscle, or MPC-myotubes receiving pro-inflammatory stimuli or not, analyzed by Western blot. The relative protein level values are expressed as a ratio [protein of interest/GAPDH or phosphorylated (P) protein/total protein]. **D** Immunofluorescence staining results of ATF6 (white arrow) in damaged TA muscle. All data are presented as means ± SD (*n* = 3). A one-way ANOVA was used for multiple comparisons (*P < 0.05; **P < 0.01). Bar = 50 μm

In CTX-induced myoinjury, transcriptome microarray and PCR analyses showed that the gene levels of CHOP, eIF2α, ATF6, ATF4, and XBP-1s in damaged muscle of WT mice were significantly elevated on day 4, after which the levels decreased on days 7 and 10 (Fig. 4A). Meanwhile, Western blot analyses showed that the ATF6, P-eIF2α, and P-IRE1α proteins were dramatically upregulated in damaged muscle until day 7, after which their expression decreased on day 10 (Fig. 4B). In line with the expression of P-eIF2α and P-IRE1α in vivo, P-eIF2α and P-IRE1α proteins were also more highly expressed in pro-inflammatory-stimulated control MPC-myotubes in vitro (Fig. 4C); however, ATF6 levels showed no changes in pro-inflammatory-stimulated MPC-myotubes when compared with controls (Fig. 4C). Consistently, we observed ATF6 staining in inflamed muscle, mainly located at the perimysium and fascicle areas (Fig. 4D), suggesting eIF2a and IRE1α arms of UPR, but not ATF6 arm, may directly modulate the protein synthesis of new myofibers in the local inflammatory microenvironment.

**Fig. 5:**
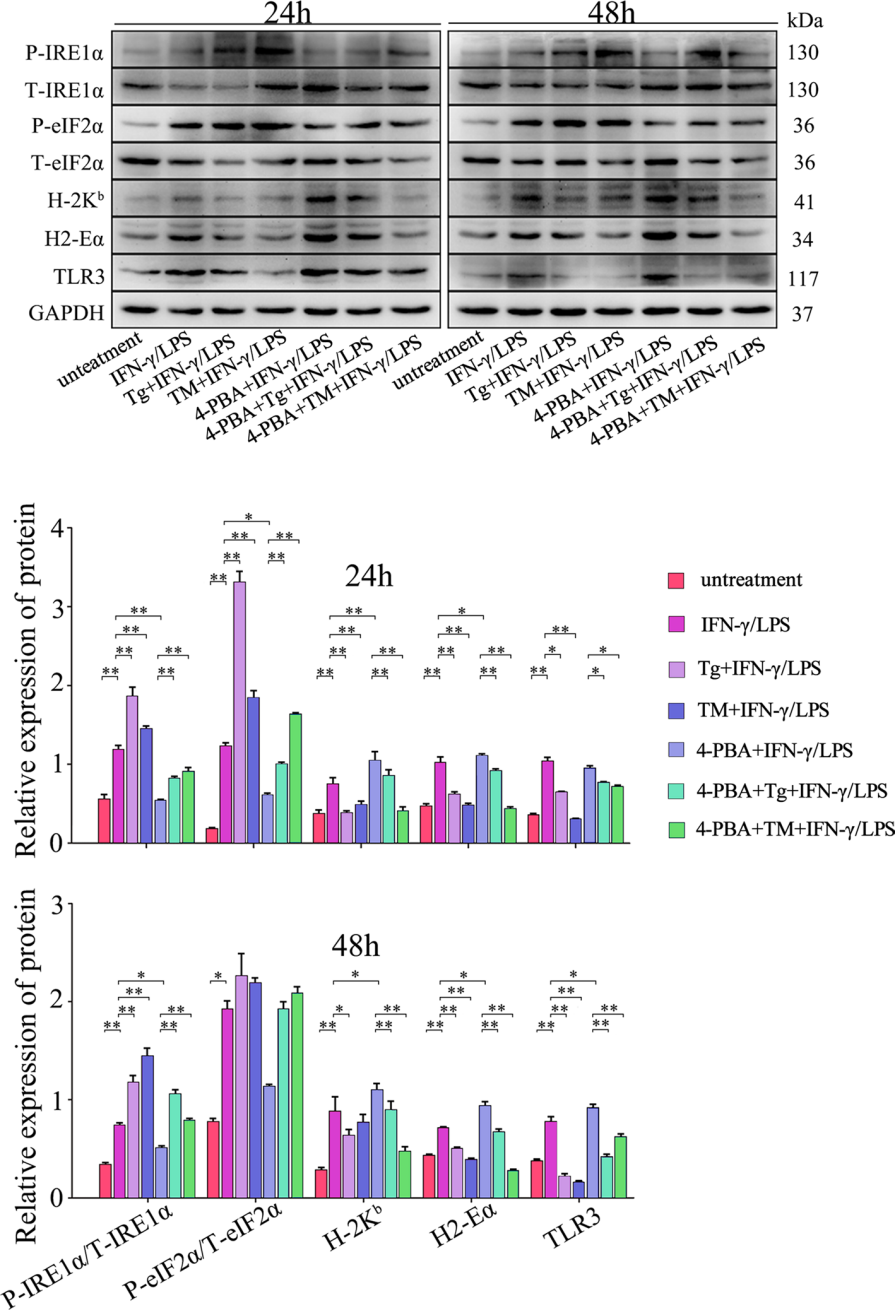
Intrinsic UPR activity affects muscle cell immune behaviors. The protein expression of the molecules related to immune response in differentiated WT mice MPC-myotubes was analyzed by Western blot. The relative protein levels are expressed as a ratio [protein of interest/GAPDH or phosphorylated (P) protein/total protein]. All data are presented as means ± SD (*n* = 3). A one-way ANOVA was used for multiple comparisons (*P < 0.05; **P < 0.01)

To determine whether the higher activity of UPR affects muscle cell capabilities for producing immune-relevant molecules, Western blot analyses were used to analyze the protein expression levels of molecules related to the immune response in differentiated MPC-myotubes. The results showed that protein levels of H-2K^b^, H2-Eα, and TLR3 decreased in groups with enhanced UPR activities after tunicamycin (TM) and thapsigargin (Tg) treatment. However, Tg-induced inhibition in expression of these immunological molecules was completely rescued by further adding the UPR inhibitor 4-PBA, resulting in a significant increase in protein expression levels of H-2K^b^, H2-Eα, and TLR3 in MPC-myotubes (Fig. 5). We monitored that 4-PBA treatment did not rescue TM-induced MHC molecule inhibition in MPC-myotubes. We speculate the distinct results of Tg and TM is due to the different induction mechanisms [36].

**Fig. 6:**
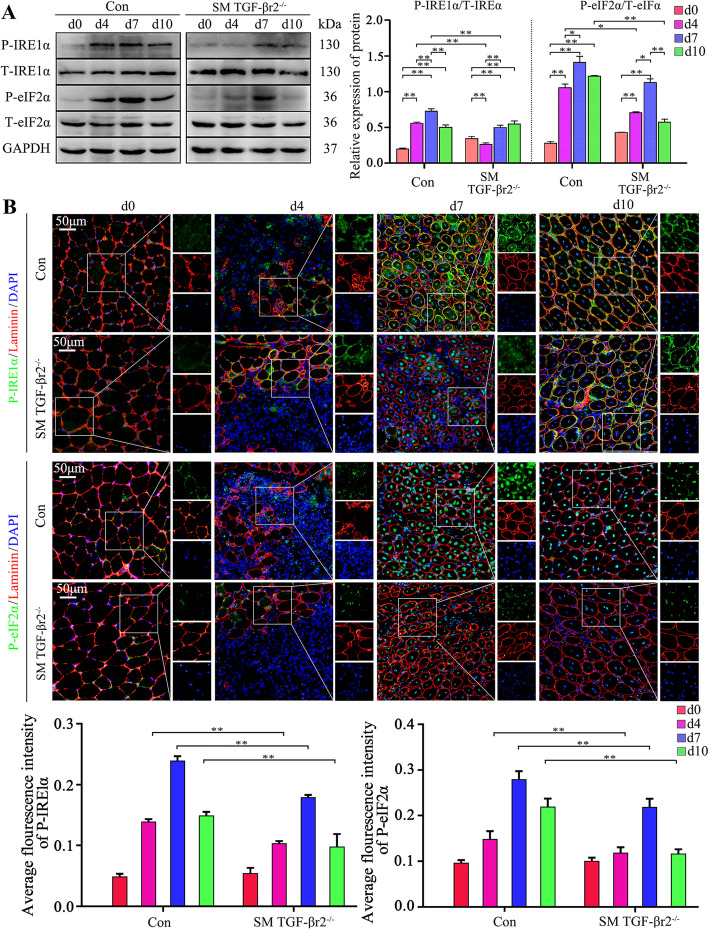
Intrinsic TGF-β signaling effects on the activities of IRE1α and eIF2α arms of UPR in muscle cell. **A** The protein levels of IRE1α and eIF2α in damaged TA muscle from control or SM TGF-βr2^−/−^ mice were analyzed by Western blot. The relative protein expression values are expressed as a ratio [phosphorylated (P) protein/total (T) protein]. **B** Immunofluorescence staining and the quantitative results of IRE1α and eIF2α in damaged TA muscle. All data are presented as means ± SD (*n* = 3). A one-way ANOVA was used for multiple comparisons (*P < 0.05; **P < 0.01). Bar = 50 μm

**Fig. 7:**
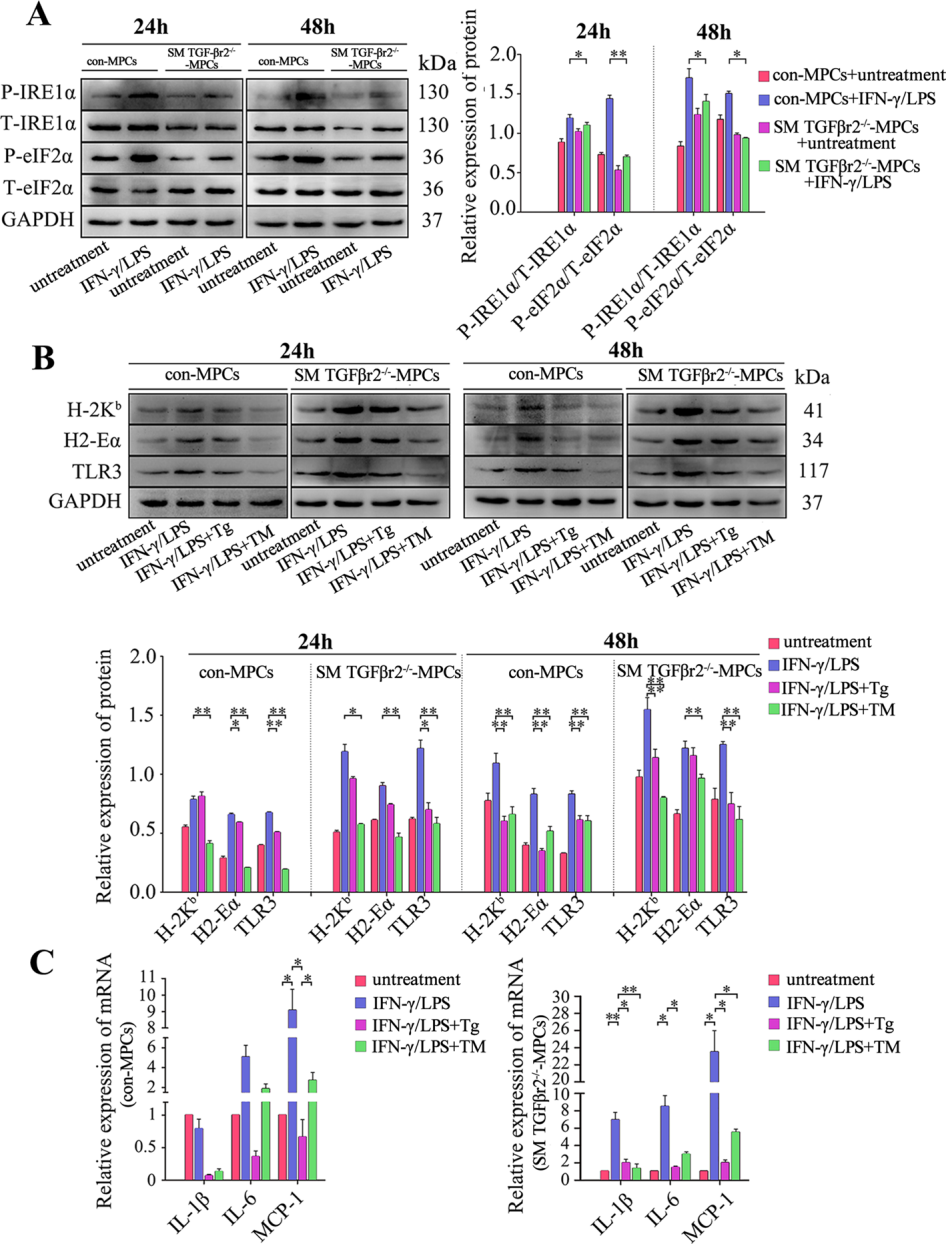
Intrinsic TGF-β signaling affects muscle cell immune behaviors by prompting UPR activity under pro-inflammatory conditions. The protein levels of IRE1α and eIF2α (**A**), H-2K^b^, H2-Eα, and TLR3 (**B**) in control or SM TGF-βr2^−/−^ mice-derived MPC-myotubes, with or without stimulation of IFN-γ/LPS, Tg, or TM, were analyzed by Western blot. The relative protein expression values are expressed as a ratio [protein of interest/GAPDH or phosphorylated (P) protein/total (T) protein]. **C** mRNA levels of IL-1β, IL-6, and MCP-1 in MPC-myotubes with or without 48 h stimulation of IFN-γ/LPS, Tg, or TM, analyzed by qPCR. All data are presented as means ± SD (*n* = 3). A one-way ANOVA was used for multiple comparisons (*P < 0.05; **P < 0.01)

Next, the SM TGF-βr2^−/−^ mice were used to clarify the relationship between TGF-β signaling and the UPR on muscle cell immunological behaviors. The results showed that on days 4 and 7 post-myoinjury, the P-IRE1α and P-eIF2α expression levels in inflamed muscle of SM TGF-βr2^−/−^ mice were distinctly lower than in control mice (Fig. 6A). It has been reported that in skeletal muscle, IRE1α-positive staining localized at the perinuclear ER and the junctional SR [37]. Our immunostaining also showed the decreased phosphorylated IRE1α and eIF2α in cytoplasm and/or nucleus of regeneration myofibers in inflamed muscle of SM TGF-βr2^−/−^ mice, when compared with control mice (Fig. 6B). In line with this result, MPC-myotubes isolated from SM TGF-βr2^−/−^ mice also displayed markedly downregulated protein expression of P-IRE1α and P-eIF2α in the presence of IFN-γ and LPS when compared with control myotubes (Fig. 7A).

**Fig. 8:**
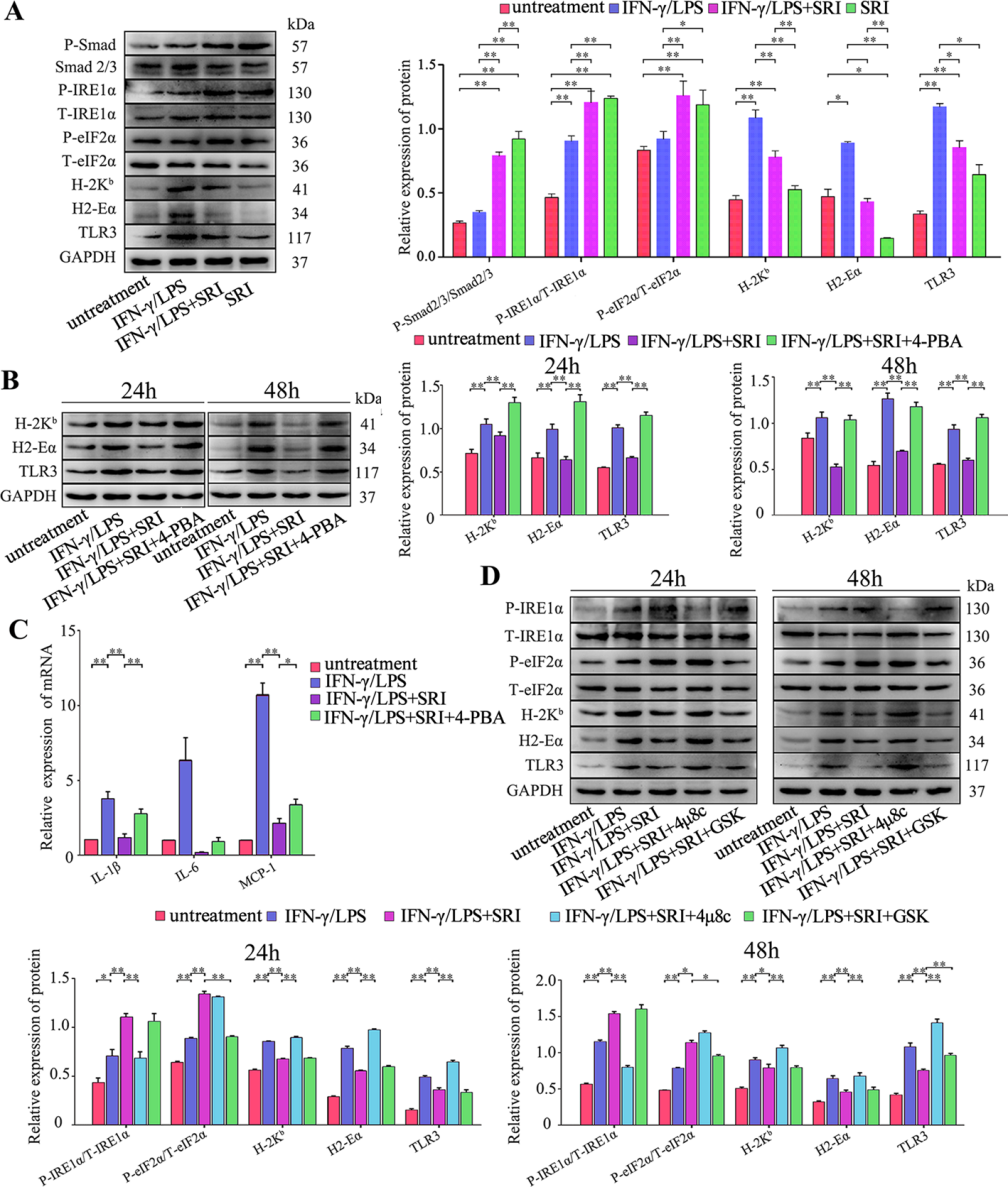
TGF-β signaling inhibits immunological characteristics of myofibers through activating the IRE1α pathway. Western blot analyses of the expression changes of Smad pathway proteins, IRE1α and PERK pathway proteins, and immunological molecules in MPC-myotubes isolated from SM TGF-βr2^−/−^ mice, with or without 48 h stimulation of IFN-γ/LPS and/or SRI (**A**); 24 or 48 h stimulation of IFN-γ/LPS, SRI, 4-PBA, 4μ8C (IRE1α pathway inhibitor) or GSK (PERK pathway inhibitor) (**B**, **D**). The relative protein expression values are expressed as a ratio [protein of interest/GAPDH or phosphorylated (P) protein/total (T) protein]. **C** mRNA levels of IL-1β, IL-6, and MCP-1 in MPC-myotubes isolated from SM TGF-βr2^−/−^ mice, with or without 48 h stimulation of IFN-γ/LPS, SRI and/or 4-PBA,analyzed by qPCR. All data are presented as means ± SD (*n* = 3). A one-way ANOVA was used for multiple comparisons (*P < 0.05; **P < 0.01)

To further identify whether the intrinsic TGF-β signal deficiency effect on muscle cell immune characteristics is due to the attenuation of UPR activity, Tg or TM was added into SM TGF-βr2^−/−^ MPC-myotube culturing system to re-initiate UPR activation in myofibers. In line with the results of LPS/IFN-γ-treated control MPC-myotubes, the administration of TM or Tg markedly decreased expression of immunobiological molecules in SM TGF-βr2^−/−^ MPC-myotubes, including TLR3, H2-Eα, and H-2 Kb proteins (Fig. 7B), and the pro-inflammatory myokines (MCP-1, IL-6, and IL-1β) gene levels (Fig. 7C). Further, when TGF-β signaling was recompensated in TGF-βr2^−/−^ MPC-myotubes through adding TGF-β/Smad agonist SRI, the results showed the highly upregulated protein expression levels of P-IRE1α and P-eIF2α (Fig. 8A). Moreover, SRI treatment effectively reversed the expression increase of immunological molecules in IFN-γ/LPS-treated TGF-βr2^−/−^ MPC-myotubes (Fig. 8A). However, the suppressed expression of immunological molecules was upregulated again by adding UPR inhibitor 4-PBA (Fig. 8B), and similarly, the gene levels of pro-inflammatory myokines were also increased in TGF-βr2^−/−^ MPC-myotubes co-treated with SRI and 4-PBA (Fig. 8C). therefore, it appears that intrinsic TGF-β signaling mediates pro-inflammatory-activated muscle cell immune capacities through prompting UPR activities.

### Intrinsic TGF-β signaling activates the IRE1α arm of UPR to downregulate the expression of muscle cell immunological molecules under an inflammatory stimulus

To further evaluate whether TGF-β signaling activates the IRE1α and eIF2α arms of UPR and simultaneously interferes with expression of immunological molecules in myofibers, IRE1α inhibitor 4μ8C (block the endonuclease activity of IRE1α) or PERK inhibitor GSK2606414 (inhibit eIF2α phosphorylation) was added to SRI-treated SM TGF-βr2^−/−^ MPC-myotubes, respectively. The results verified that, under the inflammation condition, SRI pretreated SM TGF-βr2^−/−^ myofibers presented a distinct decline in expression of H-2K^b^, H2-Eα, and TLR3, which was reversed by further treating with 4μ8C, but not with GSK2606414 (Fig. 8D). These data support that activation of IRE1α arm of UPR results in TGF-β signaling-dependent inhibition of myofiber immunological capacities.

### p38 MAPK activation is important for TGF-β-UPR-IRE1α signaling-dependent-regulation of muscle cell immunobiological capacities

**Fig. 9:**
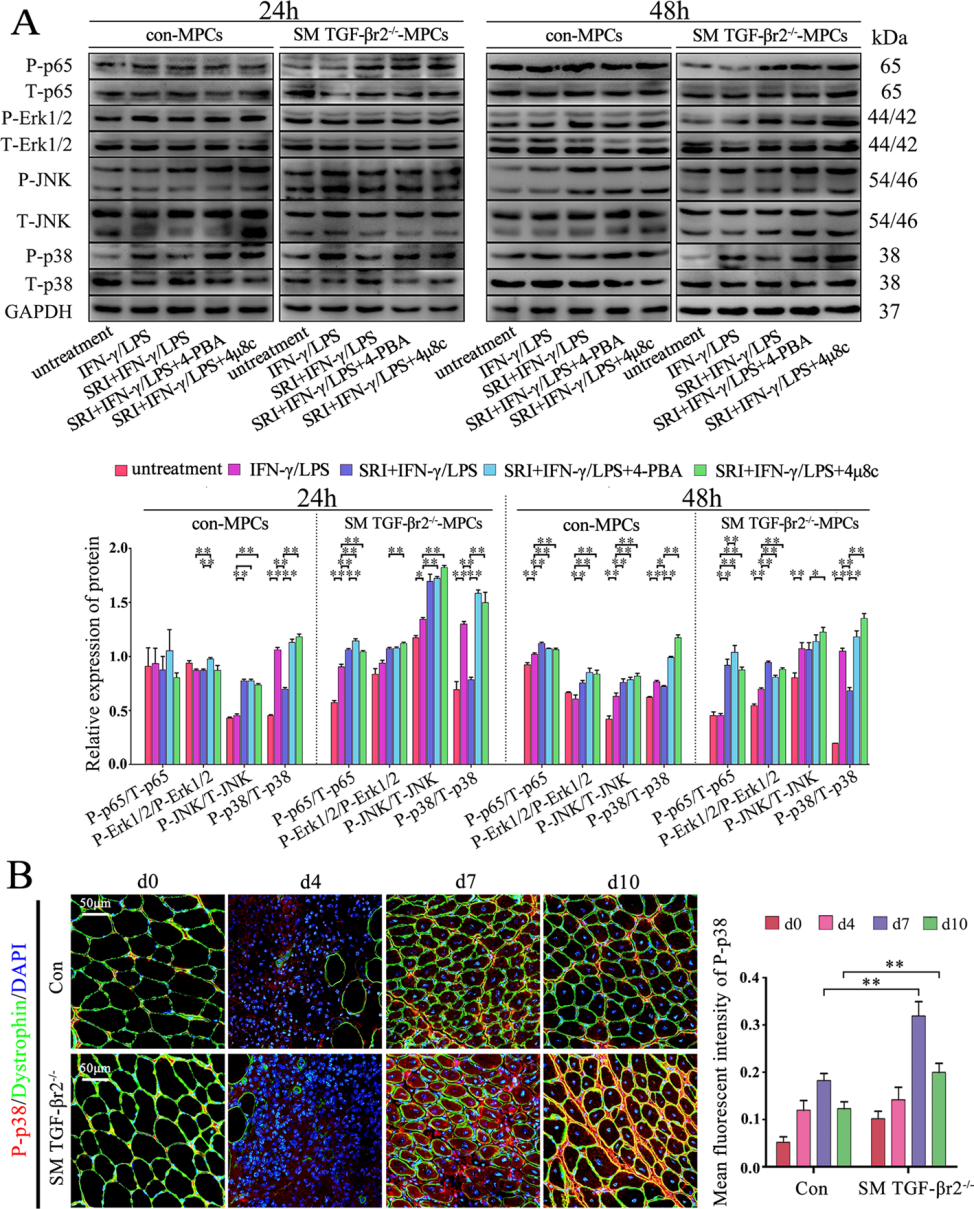
p-38 MAPk activity relates to TGF-β-IRE1α signaling in inflamed muscle cells. **A** The protein levels of Erk, Jnk, p38, and p65 in control or SM TGF-βr2^−/−^ mice-derived MPC-myotubes, with or without stimulation of IFN-γ/LPS, SRI, 4-PBA, and/or 4μ8C, analyzed by Western blot. The relative protein expression values are expressed as a ratio [phosphorylated (P) protein/total (T) protein]. **B** Immunofluorescence staining and the quantitative results of P-p38 MAPK in damaged TA muscle. All data are presented as means ± SD (*n* = 3). A one-way ANOVA was used for multiple comparisons (*P < 0.05; **P < 0.01). Bar = 50 μm

**Fig. 10:**
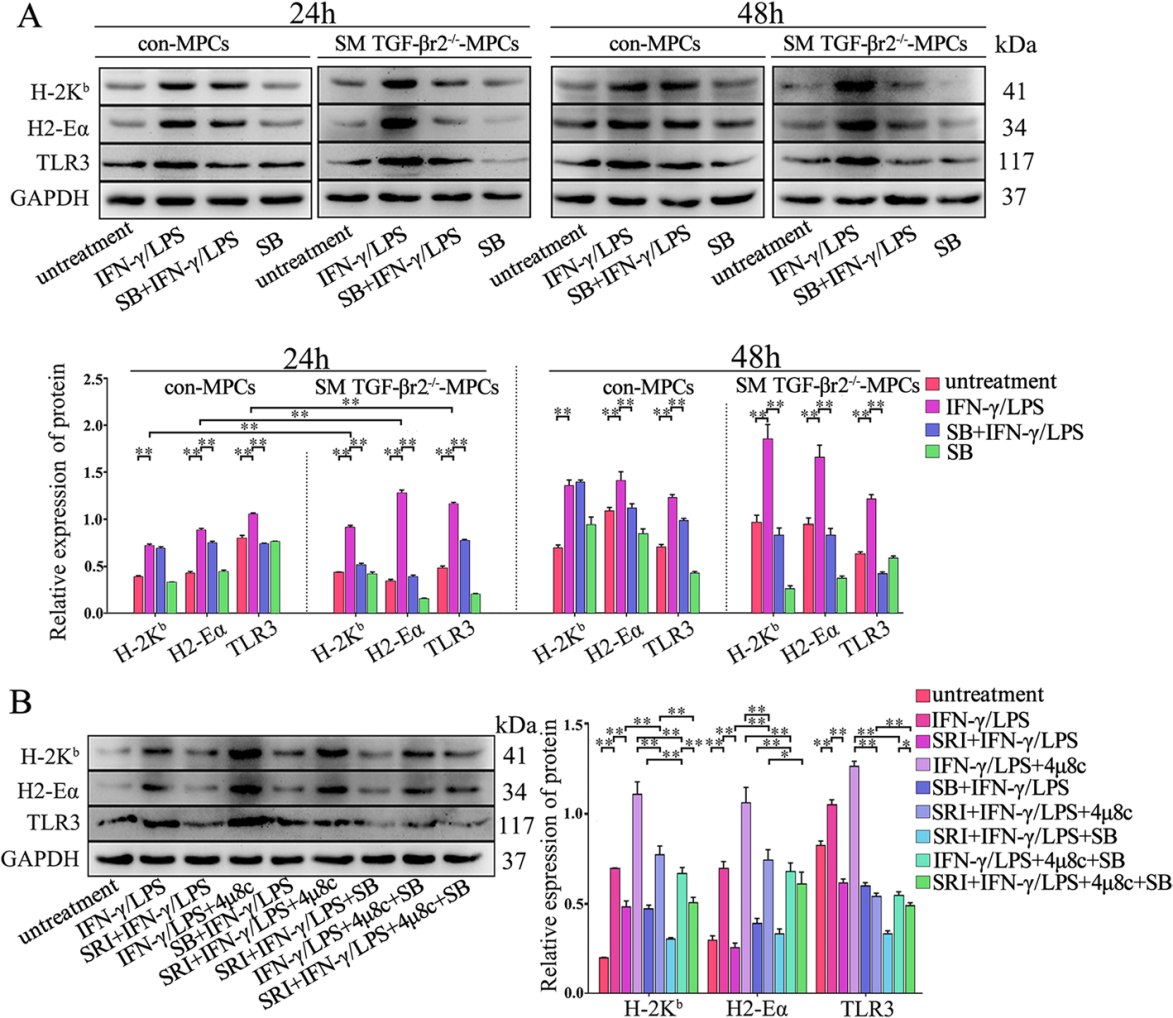
TGF-β-IRE1α signaling inhibits immunological characteristics of muscle cells by attenuating p38 MAPK pathway. Western blot analyses of the expression changes of H-2K^b^, H2-Eα, or TLR3 in MPC-myotubes derived from Con and SM TGF-βr2^−/−^ mice (**A**), or in TGF-βr2^−/−^ MPC-myotubes (**B**) with or without stimulation of IFN-γ/LPS, SRI, 4μ8C, and/or SB (p38 pathway inhibitor). The relative protein expression values are expressed as a ratio (protein of interest/GAPDH). All data are presented as means ± SD (*n* = 3). A one-way ANOVA was used for multiple comparisons (*P < 0.05; **P < 0.01)

To evaluate whether the MAPK or nuclear factor kappa-B (NF-κB) signal is involved in TGF-β-UPR signal in regulating muscle cell immunological capacities, the MAPK or NF-κB signal proteins were detected by Western blot. Compared with control MPC-myotubes, the P-p38 expression level, but not that of P-JNK, P-Erk1/2 or P-p65, was distinctly increased in LPS/IFN-γ-treated TGF-βr2^−/−^ MPC-myotubes (Fig. 9A). However, the P-p38 increase in TGF-βr2^−/−^ MPC-myotubes was reversed by adding SRI (Fig. 9A). Interestingly, when 4-PBA or 4μ8c was further added into this cell system, the P-p38 level increased again (Fig. 9A). By immunostaining, we also observed the upregulation of p38 MAPK activity in regenerating myofibers lacking TGF-βr2 (Fig. 9B). Thus, this data suggest that the function of the TGF-β-IRE1α pathway in regulating myofiber immunological capacities is possibility relevant to p-38 MAPK activity. To further verify this, TGF-βr2^−/−^ or control MPC-myotubes were treated with p38 MAPK inhibitor SB202190 (inhibiting p38 MAPK to phosphorylate) under the pro-inflammatory condition. Western blot analysis revealed that the protein level elevation of TLR3, H2-Eα, and H-2K^b^ was completely reversed by adding of SB202190 in TGF-βr2^−/−^ myofibers (Fig. 10A). Finally, adding 4μ8c corrected the decrease in expression of the immunological molecules in TGF-βr2^−/−^ MPC-myotubes treated with SRI. However, unlike the 4μ8c-treated condition, in the 4μ8c and SB202190 co-treatment condition, the decrease in levels of immunological molecules in SRI-treated SM TGF-βr2^−/−^ myofibers was not affected (Fig. 10B). Collectively, our data suggest that, under pro-inflammation stimuli, the IRE1α arm of UPR was activated in myofibers by TGF-β signaling, which then further attenuated MAPK pathways, mainly p38 signal, to suppress muscle cell immunological efficacy.

## Discussion

In various muscle immunological diseases, TGF-β signaling, ERS, and the UPR are involved in the disease pathological state [4–7, 38–40]. However, in muscle immune response, whether there is cross-talk among TGF-β signal, ERS, and UPR in regulating muscle cell immunity is unknown. This study analyzed the gene and protein expression of TGF-β2 and TGF-βr2, and the UPR pathway in CTX-damaged muscle. Moreover, to explore whether intrinsic TGF-β-UPR-IRE1α signaling affects myofiber immunobiological function through modulating the p38 MAPK pathway, analyses of the UPR and MAPK pathways, proteins of TLR3, H2-Eα, and H-2K^b^, and myokines of MCP-1, IL-6, and IL-1β were conducted in MPC-myotubes isolated from control or skeletal muscle-specific TGF-βr2 knockout mice. Our results suggest that continuously activated intrinsic TGF-β signal interferes with ERS response of myofibers under inflammatory stimulation and inhibits expression of immunological and inflammatory molecules in myofiber through the UPR-IRE1α-p38 MAPK pathway in the inflamed muscle.

Physiologically, TGF-β is produced at low levels by human and mouse immature myofibers. However, the levels of TGF-β protein are markedly elevated in inflamed myofibers, as well as in infiltrating leukocytes and endothelial cells under various myopathologies [40, 41]. The intramuscular immune response is finely regulated, and myofibers are protected from excessive inflammatory injuries by TGF-β expression [23–26, 33]. In addition, TGF-β signal controls muscle immune function through the direct modulation of adhesion molecules (e.g., ICAM-1) [23], HLA class I [24], and IL-6 secretion in myofibers [25, 26]. TGF-βr2 activation is the beginning of TGF-β signaling priming [41]. In this study, TGF-β2 and its receptor TGF-βr2 were upregulated in damaged muscle, and in MPC-myotubes, deficiency of TGF-βr2 attenuated expression of H-2K^b^, H2-Eα, TLR3, and pro-inflammatory myokines including IL-6, MCP-1, IFN-γ, RANTES, and Eotaxin. In addition, the loss of TGF-β signaling specifically in myofibers resulted in a significant increase of CD11b and F4/80 cells in CTX-damaged muscle, and impaired the transition of macrophages from M1 to M2 phenotype, suggesting the myofiber TGF-β signaling-controlled myokine production interferes with intramuscular monocyte/macrophage recruitment. Intramuscular inflammatory cells are the key regulators of muscle regeneration. Although myorepair of SM TGF-βr2^+^^+^^−/−^ mice on day 30 was comparable to that in control mice, the significantly aggravated muscle inflammation in SM TGF-βr2 mice suggests that TGF-β signaling in myofibers is important for myorepair, especially for the early myorepair process, by controlling inflammatory infiltration and guiding immune cell withdrawal after muscle damage.

Thus, our study has confirmed that the initiation of the TGF-β signaling pathway in muscle fibers participates in muscle inflammation inhibition, through controlling production of myofiber immunobiological molecules and pro-inflammatory myokines under an inflammatory stimulus.

In the immune system, the IRE1α arm of UPR plays a vital role in regulating immune cell development, differentiation, and precise function. For instance, in the condition of an acute infection, the IRE1α signal pathway was activated, which would promote T cells to differentiate into effector T cells [42, 43]. In macrophages, IRE1α activation, which may be adjusted by TLR response such as TLR2 and TLR4, may affect their function by promoting them to produce pro-inflammatory cytokines including IFN-γ, TNF, and IL-6 [44, 45]. Moreover, in dendritic cells (DCs), the IRE1α pathway also plays an important role in DC development and survival, because in XBP-1-deficient mice the detectable conventional and plasmacytoid DC numbers were decreased [45]. Accumulated MHC-I molecules in myofibers could influence the intensity of UPR, which means that the immune molecules could regulate ERS in myofibers, through which the myofiber immunological behaviors could be affected by regulating ER homeostasis in myopathic muscle [46]. Previous studies have shown that TGF-β signaling can induce IRE1α activation in different types of cells, such as hepatic stellate cells [47], cardiomyocytes [48], and endothelial cells [49]: the current study has addressed whether TGF-β could also achieve this in myofibers.

Upon pro-inflammatory milieu in vitro, our results showed that muscle fiber-specific TGF-β signaling deficiency resulted in the attenuation of UPR activity in differentiating MPCs. Of note, UPR attenuation can be corrected by adding TGF-β downstream Smad molecule SRI in TGF-βr2^−/−^ myofibers. Importantly, the data from the 4-PBA blocking of SRI-initiated UPR activation in TGF-βr2^−/−^ myofibers demonstrated that intrinsic TGF-β signaling mediates IFN-γ/LPS-activated muscle cell immunological capacities through prompting UPR activities. Further, by comparing the results from treating SRI pretreated TGF-βr2^−/−^ myofibers specifically with IRE1α inhibitor 4μ8C or PERK inhibitor GSK2606414, our study has revealed a new possible mechanism by which intrinsic TGF-β limits immune responses in myofibers, through promoting activation of the IREα pathway. Thus, in line with a previous report, our results suggest that, under a persistent pro-inflammation state, the expression levels of immunobiological molecules on regeneration myofibers are reduced by activating the IRE1α pathway, which in turn influences immune function of regenerated myofibers and the immune response of damaged muscle, and eventually affects muscle repair process.

ERS has a cross-talk with classical inflammation pathways, including NF-κB and MAPKs (p-38, JNK and Erk). For instance, JNK could be directly activated by phosphorylated IRE1α through recruiting TRAF2 and ASK1. Meanwhile, with IRE1α being continuously activated in tissues, the JNK would be activated and promote inflammation and production of pro-inflammatory chemokines [50]. The p38 MAPK could be activated at post stages of myogenesis, leading to promotion of muscle gene expression [2]. Our previous work has also noticed that p38 MAPK was notably activated by inhibiting IRE1α in myofibers with the stimulation of IFN-γ [15]. This study has further demonstrated that the upregulation of immunological molecules in myofibers, induced by 4μ8C, was reversed by inhibiting p38 MAPK, which means that p38 MAPK would have a pivotal role in regulating immunological molecules of myofibers by the IRE1α pathway. Although TGF-β-deficient myofibers can enhance IFN-γ/LPS-mediated immunobiological capacities by attenuating the activity of the IRE1α arm, whether the p38 MAPK pathway is involved in this process has previously been unclear. Here, our results show that the expression ratio of P-p65/T-p-65 and P-JNK/T-JNK in IFN-γ/LPS-treated TGF-βr2^−/−^ and control MPC-myotubes were increased compared with cells without pro-inflammatory stimuli. However, additional treatment with SRI, 4-PBA, or 4μ8C did not make any difference. On the other hand, in the absence of TGF-β signal, the expression ratio of P-p38/T-p38 considerably increased in IFN-γ/LPS-treated MPC-myotubes, and that P-p38/T-p38 increase in TGF-βr2^−/−^ MPC-myotubes was reversed by the subsequent treatment with SRI. In addition, treatment with p38 MAPK inhibitor SB202190 completely reversed the TGF-β deficiency-induced elevated expression of immunobiological molecules in myofibers exposed to IFN-γ/LPS. Together, our results have demonstrated the participation of p38 MAPK in TGF-β-IRE1α pathway in regulating myofiber immune capacities.

## Conclusions

**Fig. 11:**
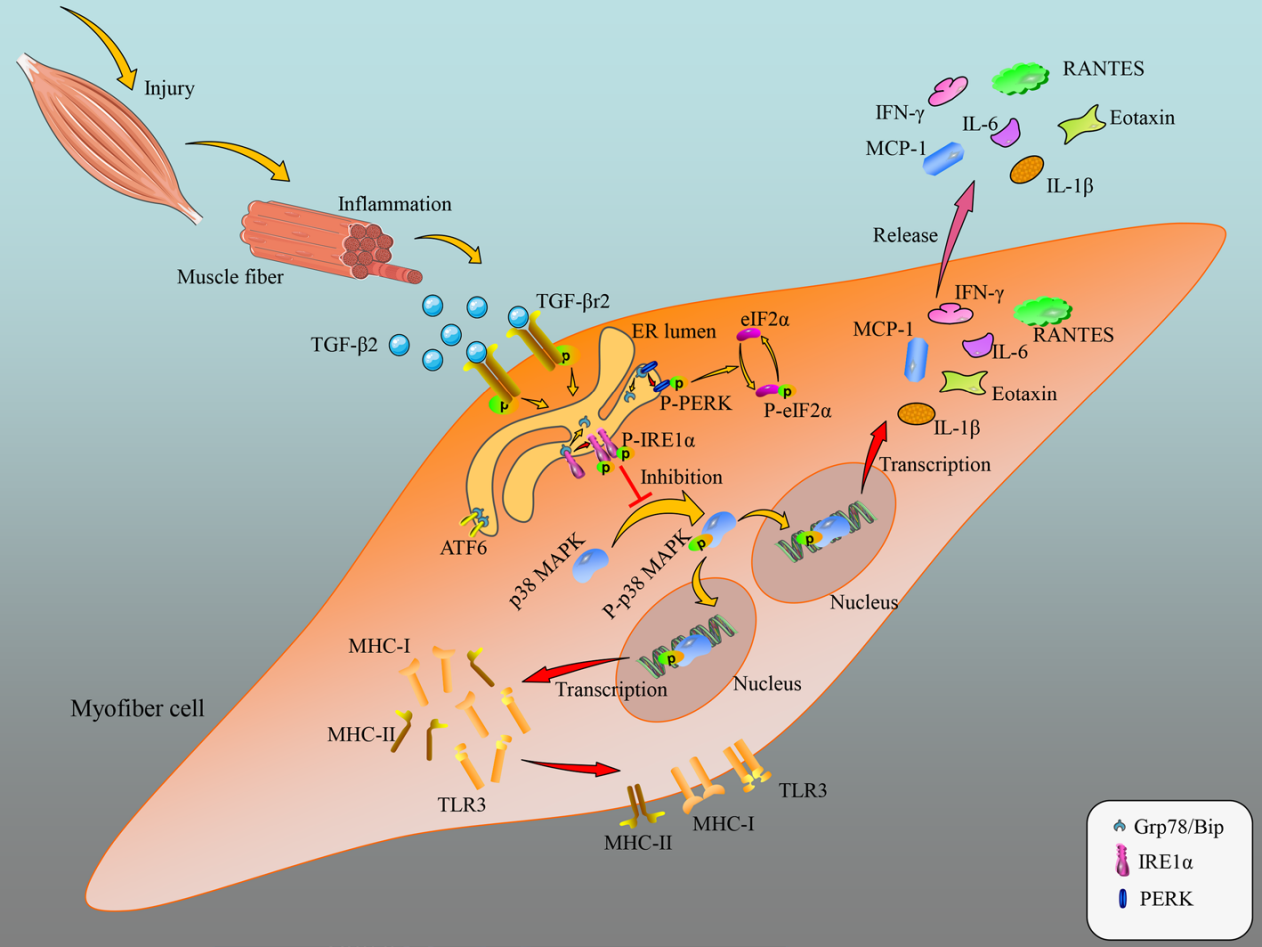
Proposed model depicting the mechanism by which TGF-β signaling acts as an upstream factor controlling myofiber immune capacities in the inflamed state by the UPR-IRE1α-p38 MAPK pathway

Combining the data from this study, we present a model depicting the mechanism by which TGF-β signaling acts as an upstream factor controlling myofiber immunological capacities in the inflamed state by impacting the UPR-IRE1α-p38 MAPK pathway (Fig. 11). In damaged muscle, the increased TGF-β2 secretes to extracellular space and binds to myofiber transmembrane TGF-βr2, activating TGF-β signaling. Subsequently, the activated TGF-β signaling promotes IRE1α phosphorylation, and the phosphorylated IRE1α then inhibits p38 MAPK activation, which eventually reduces the production of immunological molecules such as H-2K^b^, H2-Eα, and TLR3, and myokines such as eotaxin, IFN-γ, RANTES, IL-1β, IL-6, and MCP-1 in myofibers. As a result, in damaged muscle, the inflammation is attenuated, leading to regeneration and repair.

TGF-β signaling is persistently activated in myofibers under the pro-inflammatory condition, which negatively impacts upon immunological properties of muscle cells through elevating the activity of the IRE1α arm of UPR and attenuating p38 MAPK activation. This study has revealed a role and action mechanism of myofiber TGF-β signaling in regulating inflammation and repair of injured muscle. This information may assist in finding novel means for treating chronic immune myositis diseases. It is noteworthy that the degree of myocyte differentiation in vitro may affect experimental results; future work should further evaluate the role of the endogenous TGF-β-IRE1-p38 MAPK pathway on myofiber capabilities for producing immune-relevant molecules by in vivo tests.

## Supplementary Information

**Additional file 1.** The additional statistical information.

**Additional file 2: Figure S1.** SM TGF-βr2^−/−^ mice hybridization process (**A**) and their phenotype detection by PCR (**B**) and immune-staining (**C**). In PCR result, the lanes 1, 4, 7 and 9: TGF-βr2^flox/flox^/MCK-Cre^-^; the lanes 2 and 8: TGF-βr2^flox/wt^/MCK-Cre^+^; the lanes 3 and 6: TGF-βr2^wt/wt^/MCK-Cre^−^; the lane 5: TGF-βr2^wt/wt^/MCK-Cre^+^; the lanes 10 and 11: TGF-βr2^flox/flox^/MCK-Cre^+^. **Figure S2.** Myofiber TGF-β signaling deficiency impairs muscle macrophage transition from M1 to M2 phenotype and elevated gene levels of pro-inflammatory cytokines. **A** FACS analysis of the proportion of M1 (F4/80+Ly6C+) and M2 (F4/80+CD206+) cells. **B** PCR analysis of mRNA levels of pro-inflammatory mediators (IL-1β, IL-6, MCP-1 and MIP-1α) in inflamed muscle. Multiple comparisons were analyzed by One-way ANOVA. Statistical data were expressed as mean ± SD (*n* = 3). (*P < 0.05, **P < 0.01). **Figure S3.** Myofiber deficiency of TGF-β signaling had no obvious impact on myocyte regeneration. **A** Myogenin and Fast muscle myosin heavy chain (MyHC) staining. **B** Myofiber cross-sectional area (CAS) analysis. Multiple comparisons were analyzed by One-way ANOVA. Statistical data were expressed as mean ± SD (*n* = 3). Bar = 50 μm.^+^^+^^+^^+^

## Data Availability

The datasets used and/or analyzed during the current study are available from the first or corresponding author on reasonable request.

## Abbreviations

ERS: Endoplasmic reticulum stress
UPR: Unfolded protein response
MAPK: Mitogen-activated protein kinases
TGF-β: Transforming growth factor-beta
TGF-βr2: TGF-β receptor II
IRE1α: Inositol-requiring enzyme-1α
BiP: Immunoglobulin heavy chain-binding protein
XBP1s: Spliced X box-binding protein-1
PERK: Protein kinase R (PKR)-like endoplasmic reticulum kinase
HLA I: Human leukocyte antigen class I
ICAM-1: Intercellular cell adhesion molecule-1
IFN-γ: Interferon-γ
4-PBA: 4-Phenyl butyric acid
TM: Tunicamycin
TG: Thapsigargin
IL-6: Interleukin-6
T-IRE1α: Total IRE1α
H-2K^b^: Major histocompatibility complex class I molecule
H2-Ea: Major histocompatibility complex class II molecule
MyHC: Fast muscle myosin heavy chain
CTX: Cardiotoxin
MPCs: Myogenic precursor cells
PM: Polymyositis
DM: Dermatomyositis
s-IBM: Sporadic inclusion-body myositis
ECM: Extracellular matrix
SM TGF-βr2^−/−^: Muscle-specific TGF-β receptor 2 deficiency
eMHC: Embryonic myosin heavy chain
P-Jnk: Phosphorylated c-June N-terminal kinase
SRI: SRI-011381 hydrochloride
CHOP: C/EB P-homologous protein
ATF6: Activating transcription factor 6
ATF4: Activating transcription factor 4
eIF2: Eukaryotic initiation factor 2α
H&E: Hematoxylin and eosin
P-IRE1α: Phosphorylated IRE1α
TA: Tibialis anterior
TLR3: Toll-like receptors-3
DCs: Dendritic cells

## References

[1] Hetz C (2012). The unfolded protein response: controlling cell fate decisions under ER stress and beyond. Nat Rev Mol Cell Biol.

[2] Almanza A, Carlesso A, Chintha C, Creedican S, Doultsinos D, Leuzzi B, Luís A, McCarthy N, Montibeller L, More S (2019). Endoplasmic reticulum stress signalling—from basic mechanisms to clinical applications. FEBS J.

[3] Roy A, Kumar A (2019). ER stress and unfolded protein response in cancer cachexia. Cancers.

[4] Rayavarapu S, Coley W, Nagaraju K (2012). Endoplasmic reticulum stress in skeletal muscle homeostasis and disease. Curr Rheumatol Rep.

[5] Bohnert KR, McMillan JD, Kumar A (2018). Emerging roles of ER stress and unfolded protein response pathways in skeletal muscle health and disease. J Cell Physiol.

[6] Afroze D, Kumar A (2019). ER stress in skeletal muscle remodeling and myopathies. FEBS J.

[7] Bravo-Sagua R, Parra V, Muñoz-Cordova F, Sanchez-Aguilera P, Garrido V, Contreras-Ferrat A, Chiong M, Lavandero S (2020). Sarcoplasmic reticulum and calcium signaling in muscle cells: homeostasis and disease. Int Rev Cell Mol Biol.

[8] Deldicque L, Cani PD, Philp A, Raymackers JM, Meakin PJ, Ashford ML, Delzenne NM, Francaux M, Baar K (2010). The unfolded protein response is activated in skeletal muscle by high-fat feeding: potential role in the downregulation of protein synthesis. Am J Physiol Endocrinol Metab.

[9] Kim HJ, Jamart C, Deldicque L, An GL, Lee YH, Kim CK, Raymackers JM, Francaux M (2011). Endoplasmic reticulum stress markers and ubiquitin–proteasome pathway activity in response to a 200-km run. Med Sci Sports Exerc.

[10] Bohnert KR, Gallot YS, Sato S, Xiong G, Hindi SM, Kumar A (2016). Inhibition of ER stress and unfolding protein response pathways causes skeletal muscle wasting during cancer cachexia. FASEB J.

[11] Gallot YS, Bohnert KR, Straughn AR, Xiong G, Hindi SM, Kumar A (2019). PERK regulates skeletal muscle mass and contractile function in adult mice. FASEB J.

[12] Marino M, Scuderi F, Provenzano C, Bartoccioni E (2011). Skeletal muscle cells: from local inflammatory response to active immunity. Gene Ther.

[13] Ding M, Huang T, Zhu R, Gu R, Shi D, Xiao J, Guo M, Li J, Hu J, Liao H (2018). Immunological behavior analysis of muscle cells under IFN-γ stimulation in vitro and in vivo. Anat Rec.

[14] Lin YY, Belle I, Blasi M, Huang MN, Buckley AF, Rountree W, Klotman ME, Cara A, Negri D (2020). Skeletal muscle is an antigen reservoir in integrase-defective lentiviral vector-induced long-term immunity. Mol Ther Methods Clin Dev.

[15] Gu R, Huang T, Xiao J, Liao Z, Li J, Lan H, Ouyang J, Hu J, Liao H (2019). The IRE1α arm of UPR regulates muscle cells immune characters by restraining p38 MAPK activation. Front Physiol.

[16] Li Y, Foster W, Deasy BM, Chan Y, Prisk V, Tang Y, Cummins J, Huard J (2004). Transforming growth factor-beta1 induces the differentiation of myogenic cells into fibrotic cells in injured skeletal muscle: a key event in muscle fibrogenesis. Am J Pathol.

[17] Hinz B (2015). The extracellular matrix and transforming growth factor-β1: tale of a strained relationship. Matrix Biol.

[18] Lichtman MK, Otero-Vinas M, Falanga V (2016). Transforming growth factor beta (TGF-β) isoforms in wound healing and fibrosis. Wound Repair Regen.

[19] Hu HH, Chen DQ, Wang YN, Feng YL, Cao G, Vaziri ND, Zhao YY (2018). New insights into TGF-β/Smad signaling in tissue fibrosis. Chem Biol Interact.

[20] Tidball JG, Villalta SA (2010). Regulatory interactions between muscle and the immune system during muscle regeneration. Am J Physiol Regul Integr Comp Physiol.

[21] Philippou A, Maridaki M, Theos A, Koutsilieris M (2012). Cytokines in muscle damage. Adv Clin Chem.

[22] Delaney K, Kasprzycka P, Ciemerych MA, Zimowska M (2017). The role of TGF-β1 during skeletal muscle regeneration. Cell Biol Int.

[23] Marino M, Scuderi F, Mannella F, Bartoccioni E (2003). TGF-beta 1 and IL-10 modulate IL-1 beta-induced membrane and soluble ICAM-1 in human myoblasts. J Neuroimmunol.

[24] Nagaraju K, Raben N, Merritt G, Loeffler L, Kirk K, Plotz P (1998). A variety of cytokines and immunologically relevant surface molecules are expressed by normal human skeletal muscle cells under proinflammatory stimuli. Clin Exp Immunol.

[25] Mazzarelli P, Scuderi F, Mistretta G, Provenzano C, Bartoccioni E (1998). Effect of transforming growth factor-beta1 on interleukin-6 secretion in human myoblasts. J Neuroimmunol.

[26] Huang T, Huang J, Liao Z, Lan H, Jian X, Gu R, Ouyang J, Hu J, Liao H (2022). Regenerating myofiber directs Tregs and Th17 responses in inflamed muscle through the intrinsic TGF-β signaling-mediated IL-6 production. Am J Physiol Endocrinol Metab.

[27] Guardiola O, Andolfi G, Tirone M, Iavarone F, Brunelli S, Minchiotti G (2017). Induction of acute skeletal muscle regeneration by cardiotoxin injection. J Vis Exp.

[28] Hu J, Shi D, Ding M, Huang T, Gu R, Xiao J, Xian CJ, Dong J, Wang L, Liao H (2019). Calmodulin-dependent signalling pathways are activated and mediate the acute inflammatory response of injured skeletal muscle. J Physiol.

[29] Li S, Hao M, Li B, Chen M, Chen J, Tang J, Hong S, Min J, Hu M, Hong L (2020). CACNA1H downregulation induces skeletal muscle atrophy involving endoplasmic reticulum stress activation and autophagy flux blockade. Cell Death Dis.

[30] Madaro L, Marrocco V, Carnio S, Sandri M, Bouche M (2013). Intracellular signaling in ER stress-induced autophagy in skeletal muscle cells. FASEB J.

[31] Qiu L, Liu Z, Wu C, Chen W, Chen Y, Zhang B, Li J, Liu H, Huang N, Jiang Z (2020). C6-ceramide induces salivary adenoid cystic carcinoma cell apoptosis via IP3R-activated UPR and UPR-independent pathways. Biochem Biophys Res Commun.

[32] Manthey CL, Wang SW, Kinney SD, Yao Z (1998). SB202190, a selective inhibitor of p38 mitogen-activated protein kinase, is a powerful regulator of LPS-induced mRNAs in monocytes. J Leukoc Biol.

[33] Accornero F, Kanisicak O, Tjondrokoesoemo A, Attia AC, McNally EM, Molkentin JD (2014). Myofiber-specific inhibition of TGFβ signaling protects skeletal muscle from injury and dystrophic disease in mice. Hum Mol Genet.

[34] Xiong G, Hindi SM, Mann AK, Gallot YS, Bohnert KR, Cavener DR, Whittemore SR, Kumar A (2017). The PERK arm of the unfolded protein response regulates satellite cell-mediated skeletal muscle regeneration. Elife.

[35] Ruytinx P, Proost P, Van Damme J, Struyf S (1930). Chemokine-induced macrophage polarization in inflammatory conditions. Front Immunol.

[36] Abdullahi A, Stanojcic M, Parousis A, Patsouris D, Jeschke MG (2017). Modeling acute ER stress in vivo and in vitro. Shock.

[37] Wang Q, Groenendyk J, Paskevicius T, Qin W, Kor KC, Liu Y, Hiess F, Knollmann BC, Chen SRW, Tang J (2019). Two pools of IRE1α in cardiac and skeletal muscle cells. FASEB J.

[38] McLennan IS, Koishi K (1997). Cellular localisation of transforming growth factor-beta 2 and -beta 3 (TGF-beta2, TGF-beta3) in damaged and regenerating skeletal muscles. Dev Dyn=.

[39] Nagaraju K, Casciola-Rosen L, Lundberg I, Rawat R, Cutting S, Thapliyal R, Chang J, Dwivedi S, Mitsak M, Chen YW (2005). Activation of the endoplasmic reticulum stress response in autoimmune myositis: potential role in muscle fiber damage and dysfunction. Arthritis Rheum.

[40] Jackson WM, Aragon AB, Onodera J, Koehler SM, Ji Y, Bulken-Hoover JD, Vogler JA, Tuan RS, Nesti LJ (2011). Cytokine expression in muscle following traumatic injury. J Orthop Res.

[41] Murakami N, McLennan IS, Nonaka I, Koishi K, Baker C, Hammond-Tooke G (1999). Transforming growth factor-beta2 is elevated in skeletal muscle disorders. Muscle Nerve.

[42] Kemp KL, Lin Z, Zhao F, Gao B, Song J, Zhang K, Fang D (2013). The serine-threonine kinase inositol-requiring enzyme 1alpha (IRE1alpha) promotes IL-4 production in T helper cells. J Biol Chem.

[43] Pramanik J, Chen X, Kar G, Henriksson J, Gomes T, Park JE, Natarajan K, Meyer KB, Miao Z, McKenzie ANJ (2018). Genome-wide analyses reveal the IRE1a-XBP1 pathway promotes T helper cell differentiation by resolving secretory stress and accelerating proliferation. Genome Med.

[44] Shi Y, Porter K, Parameswaran N, Bae HK, Pestka JJ (2009). Role of GRP78/BiP degradation and ER stress in deoxynivalenol-induced interleukin-6 upregulation in the macrophage. Toxicol Sci.

[45] Reverendo M, Mendes A, Argüello RJ, Gatti E, Pierre P (2019). At the crossway of ER-stress and proinflammatory responses. FEBS J.

[46] Fréret M, Drouot L, Obry A, Ahmed-Lacheheb S, Dauly C, Adriouch S, Cosette P, Authier FJ, Boyer O (2013). Overexpression of MHC class I in muscle of lymphocyte-deficient mice causes a severe myopathy with induction of the unfolded protein response. Am J Pathol.

[47] Liu Z, Li C, Kang N, Malhi H, Shah VH, Maiers JL (2019). Transforming growth factor beta (TGFbeta) cross-talk with the unfolded protein response is critical for hepatic stellate cell activation. J Biol Chem.

[48] Wang Y, Zong L, Wang X (2016). TGF-beta improves myocardial function and prevents apoptosis induced by anoxia-reoxygenation, through the reduction of endoplasmic reticulum stress. Can J Physiol Pharmacol.

[49] Huang H, Ding QL, Zhu HF, Yang DF (2017). Roles of TGF-beta signaling pathway in endoplasmic reticulum stress in endothelial cells stimulated with cigarette smoke extract. J Huazhong Univ Sci Technol Med = sciences Hua zhong ke ji da xue xue bao Yi xue Ying De wen ban = Huazhong keji daxue xuebao Yixue Yingdewen ban.

[50] Bettigole SE, Glimcher LH (2015). Endoplasmic reticulum stress in immunity. Annu Rev Immunol.

