# Supplementary material for: IRE1α arm of unfolded protein response in muscle-specific TGF-β signaling-mediated regulation of muscle cell immunological properties
Source: Cell Mol Biol Lett. 2023 Feb 27;28:15. doi: 10.1186/s11658-023-00429-w (PMC9972623; doi:10.1186/s11658-023-00429-w)
Supplement: Supplementary file 1 — Additional file 1. The additional statistical information. [file 11658_2023_429_MOESM1_ESM.docx]

**The additional statistical information**

According to reviewer’s concern, we have consulted the statisticians and they point out that the high statistical significance achieved is due to that the variance of three values in each group is small enough and the difference between groups is significant. As well, the statistical methods used are reasonable and the post-hoc analyses were performed to correct for multiple comparisons in this manuscript.

For performing one way ANOVA analysis, the test of homogeneity of variance was performed. Then we check the Levene statistic result. If the result shows P>0.05, which means that the performed quantitative data is equal variances assumed, the post-hoc multiple comparisons (Least Significant Difference, LSD) were subsequently performed. On the contrary, if the equal variances not assumed (P<0.05), the Welch or Brown-Forsythe was secondly performed to test whether there is statistical significance between groups. If the robust tests of equality of means shows P<0.05, then the other post-hoc multiple comparisons (Dunnett’s T3) were finally performed.
