# Supplementary material for: IRE1α arm of unfolded protein response in muscle-specific TGF-β signaling-mediated regulation of muscle cell immunological properties
Source: Cell Mol Biol Lett. 2023 Feb 27;28:15. doi: 10.1186/s11658-023-00429-w (PMC9972623; doi:10.1186/s11658-023-00429-w)
Supplement: Supplementary file 2 — Additional file 2: Figure S1. SM TGF-βr2−/− mice hybridization process (A) and their phenotype detection by PCR (B) and immune-staining (C). In PCR result, the lanes 1, 4, 7 and 9: TGF-βr2flox/flox/MCK-Cre-; the lanes 2 and 8: TGF-βr2flox/wt/MCK-Cre+; the lanes 3 and 6: TGF-βr2wt/wt/MCK-Cre−; the lane 5: TGF-βr2wt/wt/MCK-Cre+; the lanes 10 and 11: TGF-βr2flox/flox/MCK-Cre+. Figure S2. Myofiber TGF-β signaling deficiency impairs muscle macrophage transition from M1 to M2 phenotype and elevated gene levels of pro-inflammatory cytokines. A FACS analysis of the proportion of M1 (F4/80+Ly6C+) and M2 (F4/80+CD206+) cells. B PCR analysis of mRNA levels of pro-inflammatory mediators (IL-1β, IL-6, MCP-1 and MIP-1α) in inflamed muscle. Multiple comparisons were analyzed by One-way ANOVA. Statistical data were expressed as mean ± SD (n = 3). (*P < 0.05, **P < 0.01). Figure S3. Myofiber deficiency of TGF-β signaling had no obvious impact on myocyte regeneration. A Myogenin and Fast muscle myosin heavy chain (MyHC) staining. B Myofiber cross-sectional area (CAS) analysis. Multiple comparisons were analyzed by One-way ANOVA. Statistical data were expressed as mean ± SD (n = 3). Bar = 50 μm.++++ [file 11658_2023_429_MOESM2_ESM.docx]

**Additional file 2**


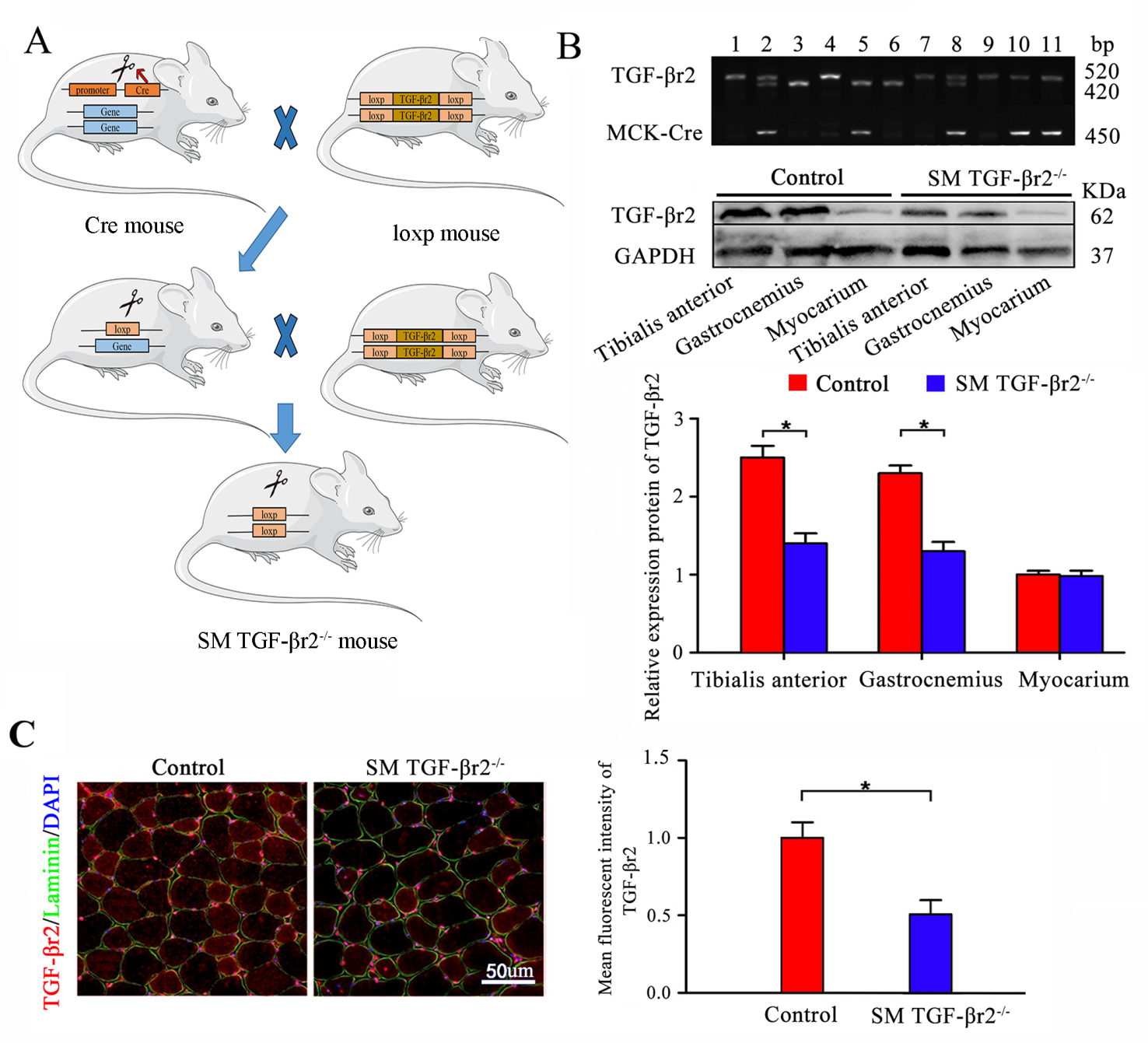


**Figure S1. SM TGF-βr2^-/-^ mice hybridization process (A) and their phenotype detection by PCR (B) and immune-staining(C).** In PCR result, the lanes 1, 4, 7 and 9: TGF-βr2^flox/flox^/MCK-Cre^-^; the lanes 2 and 8: TGF-βr2^flox/wt^/MCK-Cre^+^; the lanes 3 and 6: TGF-βr2^wt/wt^/MCK-Cre^-^; the lane 5: TGF-βr2^wt/wt^/MCK-Cre^+^; the lanes 10 and 11: TGF-βr2^flox/flox^/MCK-Cre^+^.

**
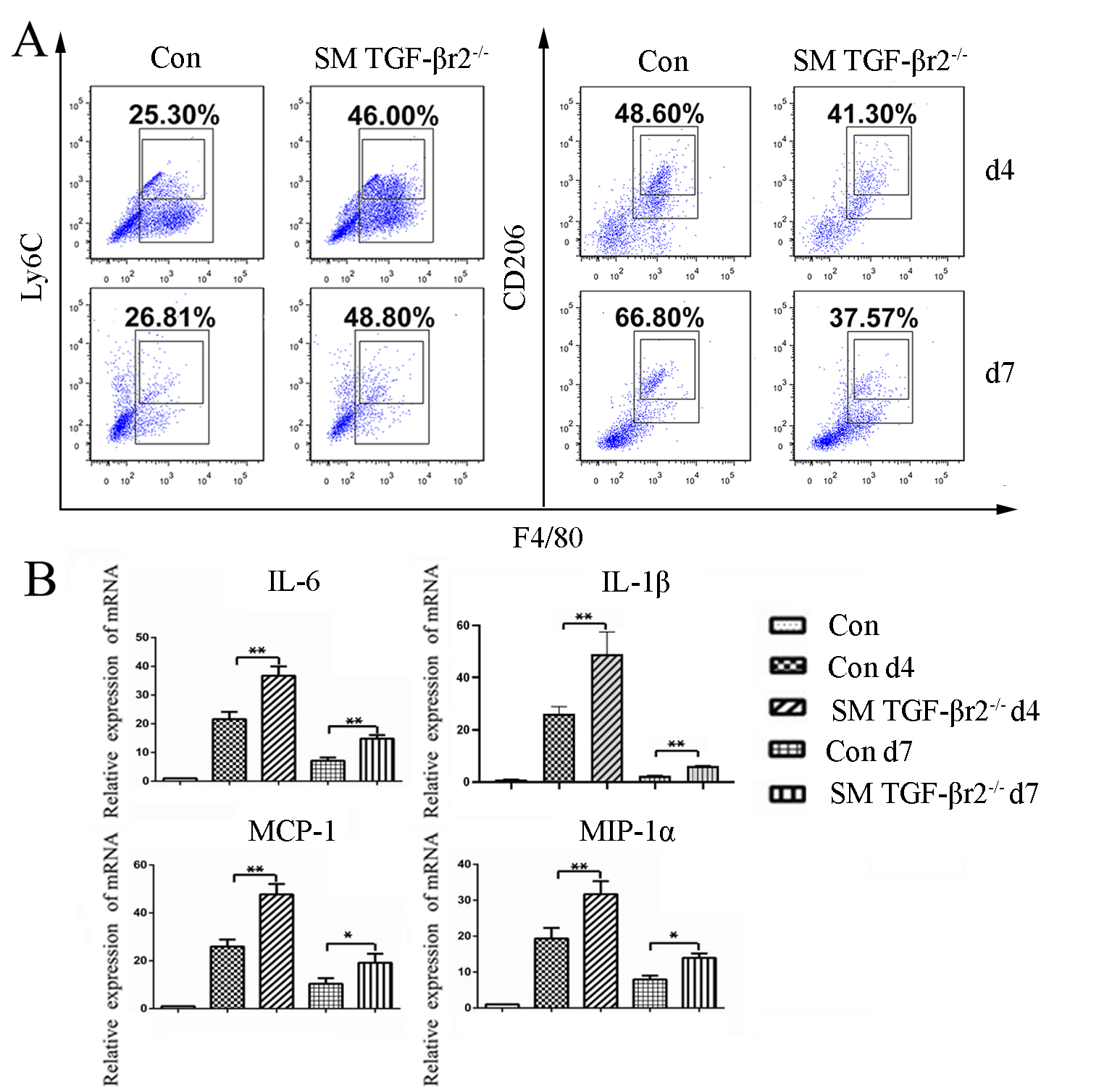
**

**Figure S2. Myofiber TGF-β signaling deficiency impairs muscle macrophage transition from M1 to M2 phenotype and elevated gene levels of pro-inflammatory cytokines.**

**A**, FACS analysis of the proportion of M1 (F4/80^+^Ly6C^+^) and M2 (F4/80^+^CD206^+^) cells. **B**, PCR analysis of mRNA levels of pro-inflammatory mediators (IL-1β, IL-6, MCP-1 and MIP-1α) in inflamed muscle. Multiple comparisons were analyzed by One-way ANOVA. Statistical data were expressed as mean±SD (n=3). (**P*<0.05, ***P*<0.01).


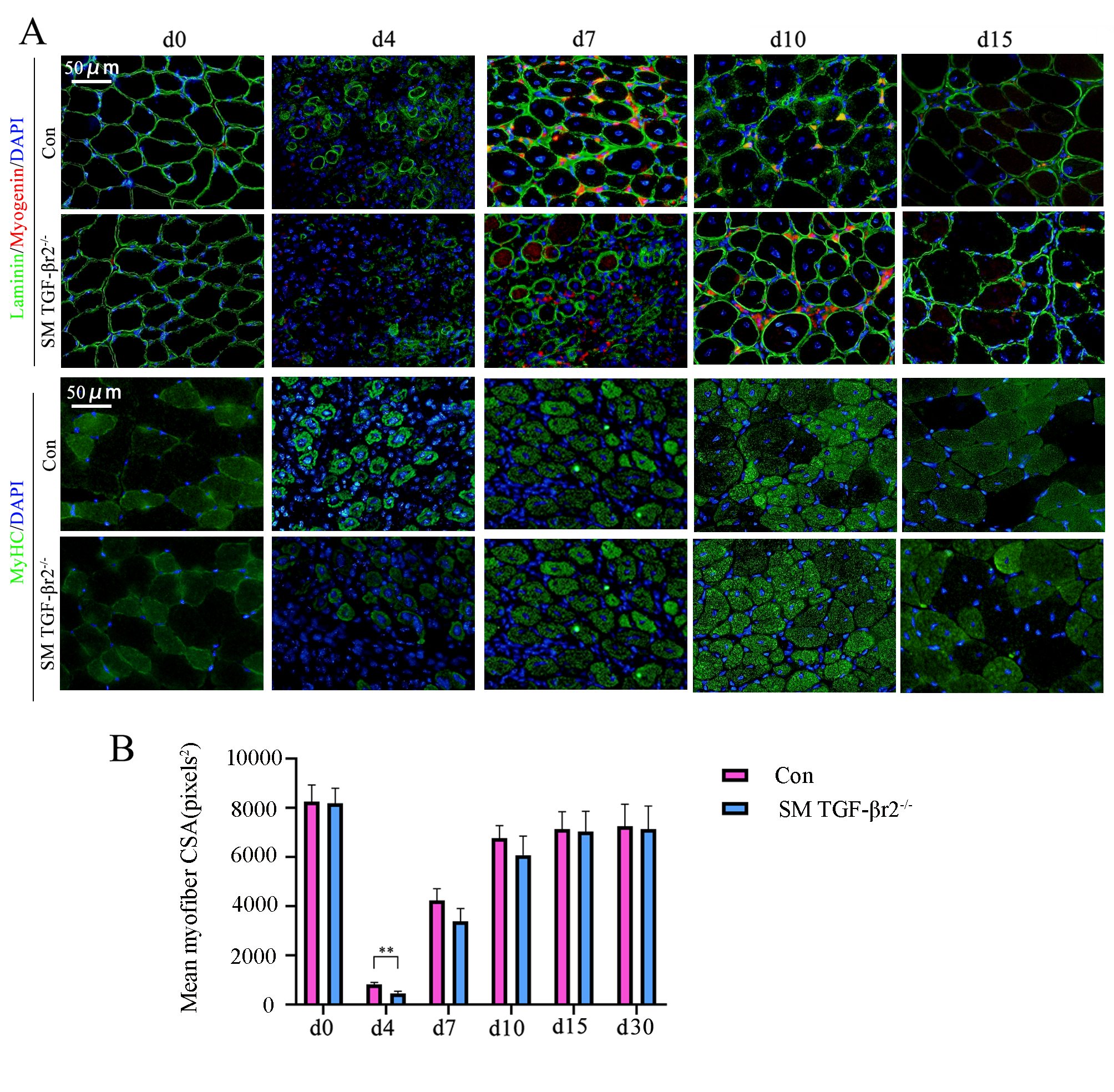


**Figure S3. Myoﬁber deficiency of TGF-β signaling had no obvious impact on myocyte regeneration.**

**A**, Myogenin and Fast muscle myosin heavy chain（MyHC）staining. **B**, Myofiber cross-sectional area (CAS) analysis. Multiple comparisons were analyzed by One-way ANOVA. Statistical data were expressed as mean±SD (n = 3). Bar = 50 μm.
